# Estimating national, regional and provincial cost-effectiveness of introducing childhood 13-valent pneumococcal conjugate vaccination in China: a modelling analysis

**DOI:** 10.1016/j.lanwpc.2022.100666

**Published:** 2022-12-19

**Authors:** Xiaozhen Lai, Cristina Garcia, Dan Wu, Maria Deloria Knoll, Haijun Zhang, Tingting Xu, Rize Jing, Zundong Yin, Brian Wahl, Hai Fang

**Affiliations:** a China Center for Health Development Studies, Peking University, Beijing, China; b Department of Health Policy and Management, School of Public Health, Peking University, Beijing, China; c Department of International Health, Johns Hopkins Bloomberg School of Public Health, Baltimore, USA; d International Vaccine Access Center, Johns Hopkins Bloomberg School of Public Health, Baltimore, USA; e National Immunization Program, Chinese Center for Disease Control and Prevention, Beijing, China; f Department of Health Management and Policy, School of Public Health, Capital Medical University, Beijing, China; g School of Public Administration and Policy, Renmin University of China, Beijing, China; h Peking University Health Science Center, Chinese Center for Disease Control and Prevention Joint Research Center for Vaccine Economics, Beijing, China; i Institute for Global Health and Development, Peking University, Beijing, China

**Keywords:** *Streptococcus pneumoniae*, Pneumococcal conjugate vaccination, China, Child health, Health economics, Economic analysis

## Abstract

**Background:**

Although 13-valent pneumococcal conjugate vaccine (PCV13) is available in China's private market, it has yet to be introduced into the National Immunization Programme (NIP) and is therefore not available to large parts of the population. This study aimed to estimate the cost-effectiveness of including PCV13 in China's NIP at national and provincial levels.

**Methods:**

We adopted a decision-tree Markov model to estimate the cost-effectiveness of adding 3-dose PCV13 in the NIP compared to the *status quo* in the private market from a societal perspective. The model hypothesized a birth cohort for five years after vaccine introduction. Treatment costs and vaccine program costs were calculated from Chinese Center for Disease Control and Prevention (CDC) and national insurance databases. Disease burden data, incidence rate ratios, and other parameters were derived from published and grey literature. Cases and deaths averted, quality-adjusted life years (QALYs) gained, and incremental cost-effectiveness ratios (ICERs) were estimated at the provincial, regional, and national levels. One-way, scenario and probabilistic sensitivity analyses were conducted to explore model uncertainty.

**Findings:**

At the national level, introducing PCV13 in the NIP was predicted to prevent approximately 4807 pneumococcal deaths (66% reduction) and 1,057,650 pneumococcal cases (17% reduction) in the first five years of the 2019 birth cohort. Under the assumed base case price of US$ 25 per dose in the NIP, PCV13 in the NIP was cost-effective nationally with ICER of US$ 5.222 per QALY gained, and was cost-effective in 17 and cost-saving in 4 of the 31 provinces compared to the *status quo*. One-way and scenario sensitivity analyses indicated robust results when varying all model parameters, and probabilistic sensitivity analysis showed a 98% probability of cost-effectiveness nationally.

**Interpretation:**

Our findings highlight the cost-effectiveness of introducing PCV13 in China's NIP. Provincial results supported subnational introduction of PCV13, and priority should be given to less socioeconomically developed provinces. Since vaccination cost is the most influential model parameter, efforts to improve PCV affordability after pooled procurement will benefit public health in a cost-effective manner.

**Funding:**

The 10.13039/100000865Bill & Melinda Gates Foundation.

**Research in context**

**Evidence before this study**

We searched PubMed, Embase, EconLit, Scopus and Web of science in English and CNKI, Wanfang and CQVIP in Chinese for national and subnational economic analyses of pneumococcal conjugate vaccine (PCV) for children under-five in China. A total of 13 articles were identified from the review, but all of them were conducted at the national level or in a single province without inter-provincial comparisons, and the majority of them focused on the cost-effectiveness of PCV7, which has been pulled from the private market in China due to the expiration of import licenses. Moreover, all studies only estimated the cost-effectiveness of PCV in the NIP compared to no vaccination, and we did not find any studies assessing the cost-effectiveness of PCV in the NIP compared to the private market nationally or at the provincial level.

**Added value of this study**

This study is the first subnational economic analysis in China to assess the cost-effectiveness of PCV13 introduction. Our estimates are based on comprehensive modelled estimates of subnational pneumococcal invasive disease morbidity and mortality in China and incorporate provincial PCV coverage in the private market. Using provincial PCV coverage rates in the private market is expected to be more precise as there are regional variations in coverage compared to the low overall coverage at the national-level. Furthermore, this is the first study to model cost-effectiveness after serotype replacement and indirect effects have stabilized using incidence rate ratio data from a global PCV impact assessment, providing more precise cost-effectiveness estimates. The Vaccine Management Law, newly released in 2019, has empowered provinces to make their own policies regarding the introduction of new vaccines, and economic estimates at the provincial level are particularly needed in China.

**Implications of all the available evidence**

Our study provides reliable subnational estimates of the cost-effectiveness of PCV13 introduction in China. The findings highlighted that PCV13 use in the NIP would be a cost-effective way to save lives and avert disability in most provinces of mainland China. The benefits could be particularly large in Qinghai, Tibet, Xinjiang and Yunnan provinces in the west region. Although these findings supported expanded access to PCV13 in the NIP, provincial-level introduction of PCV13 into local immunization programs was not only cost-effective but would also provide greater benefits in less socioeconomically developed provinces with high pneumococcal disease burden and low access to PCVs in the private market.

## Introduction

*Streptococcus pneumoniae* (pneumococcus) is a common cause of pneumonia, meningitis, bacteraemia, and acute otitis media (AOM) in young children,^1^, ^2^, ^3^ and pneumonia remains one of the leading causes of death for under-five children worldwide.^4^ As estimated by Wahl et al., China was among the ten countries with the greatest number of childhood pneumococcal deaths and cases in 2015, with approximately 7400 pneumococcal deaths and 214,800 severe pneumococcal cases nationally.^5^ A recently published domestic study revealed 8000 pneumococcal deaths and 218,200 severe pneumococcal cases annually in 2017.^6^

Preventive interventions are particularly important to prevent pneumococcal infections worldwide and mitigate a potential rise in antibiotic resistance in China.^1^^,^^7^ Vaccine-type invasive pneumococcal disease (IPD) and non-invasive pneumococcal disease in children have been reduced substantially where pneumococcal conjugate vaccine (PCV) has been used.^8^^,^^9^ The World Health Organization (WHO) recommended the inclusion of PCVs in childhood immunization programmes across the world,^1^ and as of 2021, 158 of 194 WHO member countries and regions had included childhood vaccination against pneumococcus in the National Immunization Programme (NIP).^10^ China has yet to introduce a publicly funded PCV program for young children.^7^ In China, 7-valent PCV (PCV7) and 13-valent PCV (PCV13) were regulatorily approved and made available to Chinese infants in the private market in 2008 and 2016, respectively. In 2008, China's NIP was expanded from 5 vaccines preventing 7 diseases to 14 vaccines preventing 15 diseases, but PCV7 was not included owing to shortage in domestic product supply. Later in 2015, PCV7 was pulled from the private market in China due the expiration of import licenses. Three PCV13 products are currently available in the private market in China, of which two are domestically produced and one is imported.

China continues to have a substantial burden of pneumococcal disease, and the high price of the vaccine in the private market (approximately $68–100 per dose) has restricted the widespread use of PCV nationally.^2^^,^^5^ Lack of domestic pneumococcal disease burden estimates and the high vaccination costs have impeded policymaking on the introduction of PCV into China's NIP.^7^ High-quality evidence on the health benefits and cost-effectiveness of PCV vaccination in China is limited, and provincial-level estimates are not yet available to support subnational decision-making. Economic estimates at the provincial level are particularly needed, as provinces in China are allowed to make their own vaccine introduction policies following the passage of the 2019 Vaccine Management Law. National and provincial data on the economic impact of PCV vaccination in China are needed to inform policy decisions related to expanding access to PCV. To address this research gap, we evaluated the national and provincial cost-effectiveness of introducing PCV13 into China's NIP compared to the *status quo* use of PCV13 in the private market.

## Methods

### Model overview

**Fig. 1:**
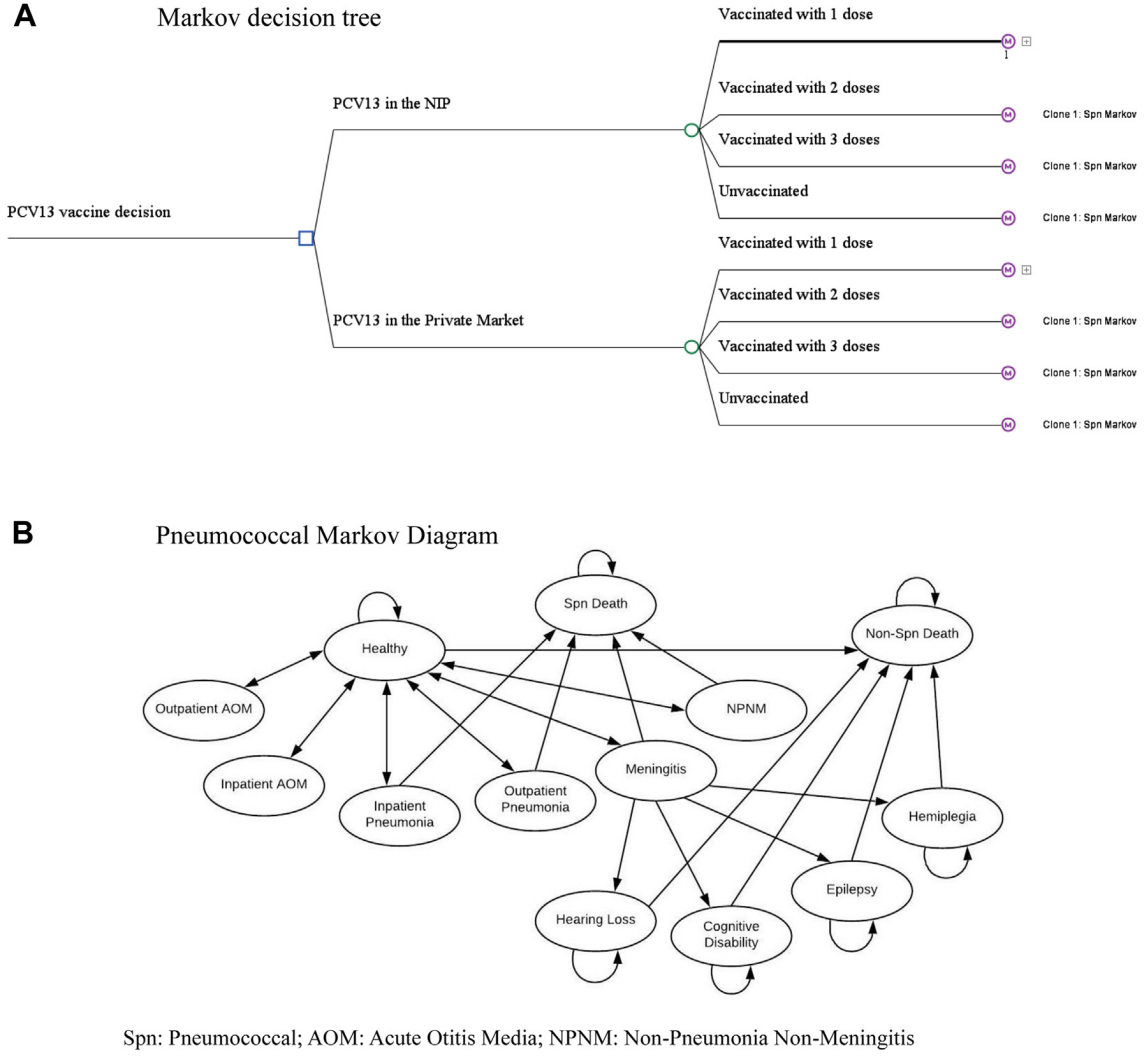
**Markov decision tree for a single birth cohort comparing PCV13 in theNationalImmunizationProgramme(NIP) vs. *status quo* (PCV13 in the private market)**. A) Markov decision tree. The Spn Markov clones have the same diagram structure (Fig. 1B), but with different probabilities due to differences in the vaccine efficacy and vaccination costs described in Table 1. B) Pneumococcal Markov Diagram. Spn: Pneumococcal; AOM: Acute Otitis Media; NPNM: Non-Pneumonia Non-Meningitis.

We developed a decision-tree Markov state transition model to access the provincial impact of PCV13 in the NIP on disease burden, quality-adjusted life years (QALYs), and costs for a hypothetical Chinese birth cohort born approximately five years after vaccine introduction when the impact of serotype replacement and indirect effects stabilized (Fig. 1). The hypothetical birth cohort's size was approximated using data from the 2019 birth cohort in China. The model compared PCV13 introduction into the NIP and the *status quo* where PCV13 was only available in the private market. We modelled PCV13 use as other PCV products were not licensed in China. Modelled health states included pneumococcal pneumonia, meningitis, non-pneumonia non-meningitis (NPNM), and acute otitis media (AOM)^1^^,^^2^^,^^6^ over the cohort's first five years of life, and QALYs gained over the life of the cohort were estimated. Children surviving past the neonatal period entered the model under both comparators (i.e., PCV13 in the NIP or *status quo*), and were assumed to be healthy but at risk of pneumococcal infections depending on vaccination status (Fig. 1).

The analysis was conducted from the societal perspective using a lifetime time horizon for the cohort. We focused on the first five years after the cohort's birth, when most costs and gains occurred, and calculated life-long losses from disease during the first five years and would not recover (i.e., premature deaths and sequelae) by discounting future losses to the birth year. All costs and effects were discounted at 3% as recommended by the WHO,^11^ and varied from 0% to 10% in one-way sensitivity analysis. All costs were converted to 2019 US dollars (1 US$ = 6.9 RMB),^12^ adjusting for inflation when necessary.^13^ The model was built in TreeAge Pro 2020 (TreeAge Software, Inc., Williamstown, MA). Results were estimated nationally and for the 31 provinces in mainland China and three geographically contiguous and socioeconomically distinct regions: east, central, and west according to National Bureau of Statistics of China. Parameter point estimates, plausibility ranges, and distributions are presented in Table 1.

**Table 1:** Province-specific model parameters and data sources.

Model parameter	Base case value	Range (min and max)	Distribution	Source
**Population at risk and demographic parameters**				
Under-five mortality rate	Province-level data	Not varied	Beta	GBD study^14^
Neonatal mortality rate	Province-level data	Not varied	Not varied	Song P et al. 2016^15^
Birth Cohort in 2019	Province-level data	Not varied	Not varied	China National Bureau of Statistics & Chinese CDC
**Epidemiologic data**				
Incidence of pneumococcal meningitis (per 100,000 children 1–59 month)	Province-level data	Webappendix 3: Table S1	Beta	Province-level disease burden model estimates^6^
Incidence of pneumococcal pneumonia (per 100,000 children 1–59 month)	Province-level data	Webappendix 3: Table S1	Beta	Province-level disease burden model estimates^6^
Incidence of pneumococcal NPNM (per 100,000 children 1–59 month)	Province-level data	Webappendix 3: Table S1	Beta	Province-level disease burden model estimates^6^
Incidence of pneumococcal AOM (per 100,000 children 1–59 month)	Province-level data	Webappendix 3: Table S1	Beta	Global Burden of Disease Study 2019 (GBD 2019) Results^16^
Age distribution				
Pneumococcal pneumonia & Pneumococcal NPNM	0–11 mo: 50% 12–23 mo: 25% 24–35 mo: 15% 36–59 mo: 10%	Not varied^a^	Not varied^a^	Watt JP et al. 2009^17^ & authors' Assumption
Pneumococcal meningitis	0–11 mo: 65% 12–23 mo: 18% 24–35 mo: 7% 36–59 mo: 10%	Not varied^a^	Not Varied^a^	Watt JP et al. 2009^17^ & authors' Assumption
Pneumococcal AOM	0–11 mo: 21.3% 12–59 mo: 78.7%	Not varied^a^	Not Varied^a^	Global Burden of Disease Study 2019 (GBD 2019) Results^16^
Case fatality ratios				
Pneumococcal pneumonia	Province-level data	Webappendix 3: Table S1	Beta	Province-level disease burden model estimates^6^
Pneumococcal meningitis	Province-level data	Webappendix 3	Beta	Province-level disease burden model estimates^6^
Pneumococcal NPNM	Province-level data	Webappendix 3	Beta	Province-level disease burden model estimates^6^
Meningitis neurological sequelae	1.40%	1.05–1.75%	Beta	Eke CB et al. 2016^18^
Meningitis sequelae				
Probability of cognitive disability	1.60%	1.0%–1.3%^b^	Beta	Edmond K et al. 2010^19^
Probability of epilepsy	2.20%	2.1%–3.2%^b^	Beta	Edmond K et al. 2010^19^
Probability of hemiplegia	3.20%	2.2%–8.1%^b^	Beta	Edmond K et al. 2010^19^
Probability of hearing loss	4.60%	3.1%–8.2%^b^	Beta	Edmond K et al. 2010^19^
Probability of cochlear implant	40.00%	30.0%–50.0%^b^	Beta	Sun B et al. 2015^20^
Probability of special education	65.00%	48.8%–81.3%^b^	Beta	2018 China Education Statistics Yearbook^21^
**Vaccine efficacy & coverage**				
1-dose efficacy for IPD	62.00%	46.5%–77.5%	Beta	Lucero MG et al. 2009^22^ & Authors' assumption
2-dose efficacy for IPD	80.00%	58.0%–90.0%	Beta	Lucero MG et al. 2009^22^
3-dose efficacy for IPD	80.00%	58.0%–90.0%	Beta	Lucero MG et al. 2009^22^
4-dose efficacy for IPD	80.00%	58.0%–90.0%	Beta	Assumed by 3-Dose Efficacy
1-dose efficacy for AOM	21.00%	0%–65.0%	Beta	Eskola J et al. 2001^23^
2-dose efficacy for AOM	43.00%	0%–71.0%	Beta	Eskola J et al. 2001^23^
3-dose efficacy for AOM	57.00%	36.0%–72.0%	Beta	Eskola J et al. 2001^23^
4-dose efficacy for AOM	57.00%	36.0%–72.0%	Beta	Assumed by 3-Dose Efficacy
PCV13 coverage in private market	Province-level data	Webappendix 5: Table S2	Triangular	Chinese CDC
PCV13 coverage in national immunization program	Region-level data	Webappendix 5: Table S3	Triangular	Assumed by DTP vaccine coverage rate in China^15^
Incidence rate ratio of PCV-13 for IPD	0.3334	0.2534–0.4386	Normal	Deloria Knoll M. 2021^24^
Incidence rate ratio of PCV-13 for AOM	0.8660	0.6495–1.0000	Normal	Marom T et al. 2021^9^
**Cost of illness (USD)**				
Cost per inpatient pneumonia case	Province-level data	Webappendix 4: Table S4	Gamma	CHIRA,^25^ Chinese CDC & 2020 China Statistics Yearbook^26^
Cost per inpatient meningitis case	Province-level data	Webappendix 4: Table S5	Gamma	CHIRA,^25^ Chinese CDC & 2020 China Statistics Yearbook^26^
Cost per inpatient NPNM case	Province-level data	Webappendix 4: Table S5	Gamma	CHIRA,^25^ Chinese CDC & 2020 China Statistics Yearbook^26^
Cost per inpatient AOM case	Province-level data	Webappendix 4: Table S6	Gamma	CHIRA,^25^ Chinese CDC & 2020 China Statistics Yearbook^26^
Cost per outpatient pneumonia case	Province-level data	Webappendix 4: Table S4	Gamma	CHIRA,^25^ Chinese CDC & 2020 China Statistics Yearbook^26^
Cost per outpatient AOM case	Province-level data	Webappendix 4: Table S6	Gamma	CHIRA,^25^ Chinese CDC & 2020 China Statistics Yearbook^26^
Cost per cognitive disability case	1302	976–1627	Gamma	CHIRA^25^
Cost per hearing loss case	3636	2727–4545	Gamma	CHIRA^25^
Cost per epilepsy case	823	617–1029	Gamma	CHIRA^25^
Cost per hemiplegia case	1734	1301–2168	Gamma	CHIRA^25^
Cost of cochlear implant per case	18,715	14,036–23,394	Gamma	Qiu J et al. 2017^27^
Discounted cost of special education (age 6–18)	Province-level data	Webappendix 4: Table S2	Gamma	2018 China Education Statistics Yearbook^21^ & 2018 China Education Expenditure Statistics Yearbook^28^
Discounted lifetime productivity per capita	Province-level data	Webappendix 4: Table S2	Gamma	2010 China census data^29^
**Utilities**				
Utility of meningitis	0.9768	0.5970–1	Beta	Bennett JE et al. 2010^30^
Utility of outpatient pneumonia or NPNM	0.9963	0.9926–1	Beta	van Hoek AJ et al. 2012^31^
Utility of inpatient pneumonia	0.9941	0.7948–1	Beta	Bennett JE et al. 2010^30^
Utility of inpatient NPNM	0.9921	0.7825–1	Beta	Bennett JE et al. 2010^30^
Utility of AOM	0.995	0.9341–1	Beta	Oh PI et al. 1996^32^
Utility of cognitive disability	0.62	0.51–0.73	Beta	Oostenbrink R et al. 2002^33^
Utility of hearing Loss	0.91	0.83–1	Beta	Oostenbrink R et al. 2002^33^
Utility of epilepsy	0.83	0.75–0.91	Beta	Oostenbrink R et al. 2002^33^
Utility of hemiplegia	0.3903	0–1	Beta	Bennett JE et al. 2010^30^
**Immunization costs (USD)**				
Vaccine price per dose in the private market	68.12	51.09–85.14	Gamma	Chinese CDC
Vaccine price per dose in the private market	25.00	18.75–31.25	Gamma	Upper bound recommended by UNICEF for MICs^34^
Cost of immunization delivery per dose	Province-level data	Webappendix 5: Table S4	Gamma	Chinese CDC^35^
Incidence of PCV13 severe adverse events (per 100,000 doses)	1.24	0–89.52	Beta	Li K et al. 2020^36^
Cost of adverse events per case	935	702–1169	Gamma	2020 China Health Statistics Yearbook^37^
Wastage rate	5.00%	0.0%–10.0%	Beta	Authors' assumption

NPNM Non-Pneumonia Non-Meningitis.

GBD Global Disease Burden.

CHIRA China Health Insurance Research Association.

The base case values and ranges of all province-level data are presented in corresponding Webappendix.

Webappendix 2: Population at risk and demographic parameters.

Webappendix 3: Epidemiologic data.

Webappendix 4: Costs of Illnesses.

Webappendix 5: PCV13 coverage and Immunization costs.

a Parameter not varied in the sensitivity analyses. Age distribution uncertainty is included in the disease burden incidence uncertainty.

b 95% Confidence Interval (CI).

### Epidemiological data

Given the technical challenges of doing observational studies for pneumococcal disease burden based on surveillance systems,^38^ there were no directly measured burden estimates from China, but modelled estimates could help support decision-making in the absence of empirical data. The provincial-level age-specific probabilities of IPD cases and deaths, including pneumococcal severe and non-severe pneumonia, meningitis and NPNM, were estimated from modelled 2019 provincial pneumococcal incidence and mortality estimates assuming no vaccination in the private market (Webappendix 3: Table S1).^6^ We assumed all severe pneumonia, meningitis, and NPNM cases were hospitalized, and all deaths occurred in hospitals due to the high access to care across China. Children developing pneumococcal meningitis in the model were at risk of long-term sequelae (i.e., cognitive disability, hearing loss, epilepsy and hemiplegia) following probabilities from a global meta-analysis study.^19^ In addition to IPD, pneumococcus could also cause non-invasive disease among which AOM causes the largest disease burden. We obtained age-specific AOM incidence estimates for children aged 1–59 months in China in 2019 prepared by the Global Burden of Disease, Injuries, and Risk Factors Study (GBD).^14^ Because AOM rarely causes death, AOM deaths were not considered in the present study.

### Economic costs of diseases

Provincial and regional costs of pneumococcal disease by illness states were estimated from published literature and health insurance data from the China Health Insurance Research Association (CHIRA), which comprised individual-level data from hospitals in all 31 provinces in Mainland China between 2013 and 2017.^25^ The cost per inpatient and outpatient case of pneumonia, meningitis, NPNM and AOM included direct medical, direct non-medical and indirect costs. The cost of sequelae included direct medical costs, indirect future productivity loss, costs for possible cochlear implant after hearing loss, and costs for special education.

We calculated the average direct medical cost per case for each syndrome using ICD-10 codes from CHIRA database. Direct non-medical costs of inpatient pneumonia and meningitis cases were retrieved from two Chinese CDC surveys conducted in Gansu, Hubei, Shandong and Hebei provinces.^39^^,^^40^ Provincial estimates were calculated by adjusting the survey estimates to the ratio of provincial total consumption expenditures reported by the China Statistics Yearbook.^26^ Direct non-medical costs of NPNM and AOM cases were obtained by multiplying the average hospitalization stay and daily non-medical cost for inpatient pneumonia due to lack of reliable data from literature. Human capital approach was adopted to estimate the indirect cost of caregivers' and visitors’ productivity loss and future lifetime productivity loss due to premature death and disability. The provincial-level direct medical, non-medical and indirect costs for each pneumococcal syndrome were in Webappendix 4: Tables S3 and S6.

### Vaccine efficacy and coverage rates

Although a 4-dose schedule is currently used in the private market, no decision has been made on a uniform PCV schedule for the NIP. The base case modelled a 3-dose schedule (3 + 0) following the recommendation of the latest WHO position paper.^1^ For the *status quo* scenario, provincial coverage rates for each PCV13 dose in the private market were estimated by multiplying the total doses administered in each province from the Chinese CDC by the distribution of children receiving 1, 2, 3, and 4 doses obtained from a 2019 facility-based survey of more than 6000 children in 10 provinces in China (Webappendix 5: Table S2).^41^ For vaccines included in the NIP, vaccination was required for children prior to school entry, and the regional 3-dose diphtheria-tetanus-pertussis vaccine (DTP) coverage was used as a proxy for PCV13 coverage in the NIP because of the similar schedule in China. Dose-specific vaccine efficacy against IPD was estimated from a meta-analysis of controlled clinical trials globally,^22^ and that of AOM was retrieved from a randomized, double-blind efficacy trial.^23^

Recent analyses of data from countries with multiple years of pre- and post-PCV introduction surveillance showed that incidence rates of vaccine-serotypes and non-vaccine-serotypes fluctuated due to herd effects and serotype replacement as vaccine coverage scaled up. A recent study evaluating the impact of serotype replacement estimated that the combined effects stabilized about five years after introduction.^24^ To provide results that could reflect the cost-effectiveness once coverage and vaccine effects stabilized after the introduction of PCV13 in the NIP, we modelled the costs and effects five years after introduction using the pre- and post-PCV13 introduction incidence rate ratios (IRR), instead of using merely vaccine efficacy as done in the *status quo* scenario. The IRR measure accounted for the combined impact of vaccine coverage, herd effects, and serotype replacement. The IRR for IPD was estimated from the PSERENADE global meta-analysis,^24^ and that of AOM was obtained from a population-based study.^9^ See Table 1 for the IRR used as well as provincial and regional weighted vaccine coverage estimates.

### Vaccination costs

In China, two domestically manufactured PCV13 products were approved and available in the private market for children under-five (Webappendix 5) at the lowest price of US$ 68.12 per dose in 2021. The base case modelled a 3-dose schedule (3 + 0) for both strategies following the recommendation of the latest WHO position paper,^1^ but because the price per dose was likely to decline from large-scale procurement, we assumed a base case price of US$ 25 per dose in the NIP, which was the upper range of UNICEF recommended price for middle-income countries (MICs).^34^ The societal cost of the PCV13 program, including the governmental cost of routine immunization and the household cost of vaccine-seeking, was estimated using regional vaccine program data from a 2015 survey conducted by the Chinese CDC in 15 provinces.^35^ Governmental costs included the cost of vaccines, wastage, personnel, cold chain, surveillance, communication activities, training, supervision at the national and provincial levels and the cost of serious adverse reaction. Vaccine-seeking costs included the cost of transportation and caregiver productivity loss. See Webappendix 5 for the methods used to estimate the societal costs of the vaccine program for each strategy.

### Cost-effectiveness analysis

Incremental cost-effectiveness ratios (ICERs), defined as the incremental costs (i.e., PCV13 costs and disease costs) per pneumococcal case averted, per death averted and per QALY gained, were used to compare the *status quo* and NIP strategies. The length of the Markov cycle was one year, and the cohort was followed in the first five years of life since the immunity of PCV13 could persist over this period and most pneumococcal cases would occur in under-five children.^6^ We derived utilities of health states from previous literature in the United States, Canada and Netherlands^30^, ^31^, ^32^, ^33^ since China lacked domestic measures on utility values of childhood pneumococcal disease. QALY utilities ranged from 0 to 1, where 0 represented death and 1 represented perfect health (Table 1). The Chinese government did not set any thresholds for assessing the cost-effectiveness of vaccines, so we adopted two widely-acknowledged thresholds: 1) national and provincial GDP per capita as recommended by the Commission on Macroeconomics and Health^42^; and 2) threshold estimated by Cai et al. for China (2019 US$ 14,897), which used a modified value of statistical life approach.^43^ The 2019 national GDP per capita was US $10.274, and provincial GDPs were described in Webappendix 2: Table S1.^26^

### Sensitivity analysis

We further conducted sensitivity analyses to measure the uncertainty and robustness of model results. Deterministic sensitivity analyses (DSA) were done at the national level by applying a range of plausible values for each parameter entered into the model (see Table 1). In the absence of uncertainty ranges, including 95% confidence intervals, from the parameter source, the plausibility range was assumed to be ±25% of the base value. Probabilistic sensitivity analysis (PSA) using Monte Carlo simulation (N = 10,000 iterations) was also performed to assess the effects of simultaneously changing multiple parameters. Model uncertainty from the DSA was summarized using a tornado diagram, and the PSA was used to generate cost-effectiveness acceptability curves at the national level.

Scenario sensitivity analyses were performed to adjust the vaccine price per dose and vaccine schedules in the NIP. To estimate the influence of the NIP vaccine price on cost-effectiveness, we estimated the cost-effectiveness when keeping the price per dose of PCV13 in the NIP the same as the current price in the private market (US$ 68.12). Additionally, because the government would negotiate the price per dose and the size of China's birth cohort was larger than the birth cohort in all of Latin America, we estimated the cost-effectiveness when the NIP price was the same as the Pan American Health Organization (PAHO) price (US$ 14.5). We also estimated the cost-effectiveness of other scenarios to help inform decision-making, including a 3 + 1 schedule as recommended by vaccine manufacturers, IPD IRR ranging from 0.2 to 0.5, and expected 3-dose coverage rates at different levels.

### Role of the funding source

The sponsor of this study had no role in study design, data collection, data analysis, data interpretation, writing of the report, or the decision to submit for publication.

## Results

### Impact of PCV13

Table 2 presented the projected health effects and costs of including PCV13 in the NIP for the hypothetical birth cohort in China. Compared with the *status quo*, PCV13 in the NIP was estimated to avert approximately 1,057,650 pneumococcal cases (17% reduction) and 4.807 pneumococcal deaths (66% reduction) over the first five years of life for the cohort. Most averted cases were AOM (69.1%), and most averted deaths were inpatient pneumonia (66.2%) (Webappendix 6: Table S1). The greatest number of cases averted (i.e., >100,000 cases) happened in Guangdong, the most populous province in China, and most deaths averted (i.e., >500 deaths) happened in Yunnan, where the PCV13 coverage in the private market was low and the case fatality was high. The NIP strategy resulted in 146,335 QALYs gained over the cohort's lifetime with most QALYs gained in provinces in the west region and poorer provinces like Yunnan.

**Table 2:** Estimated cases, deaths and QALYs averted and ICERs over the first five years of life by province for PCV13 in the NIP compared with PCV13 in the private market.

Province and Region	Status Quo				NIP				Difference				ICERs		
	Total Spn Cases	Total Spn Deaths	QALYs	Total Costs (US$)	Total Spn Cases	Total Spn Deaths	QALYs	Total Costs (US$)	Cases Averted	Deaths Averted	QALYs Gained	Incremental Costs (US$)	US$ per Case Averted	US$ per Death Averted	US$ per QALY Gained
**Anhui**	223,332	222	18,879,640	42,879,950	184,472	74	18,884,165	81,959,953	38,860	147	4526	39,080,003	1006	264,974	8635
**Beijing**	122,896	60	6,677,701	32,975,386	103,299	21	6,678,930	41,433,372	19,597	39	1229	8,457,986	432	218,382	6879
**Chongqing**	115,693	95	8,627,296	24,522,656	95,680	32	8,629,239	41,954,146	20,013	63	1943	17,431,490	871	277,961	8971
**Fujian**	164,045	134	14,120,023	33,968,194	136,058	45	14,122,774	67,028,148	27,986	89	2751	33,059,954	1181	370,568	12,018
**Gansu**	134,139	290	9,238,231	39,707,137	110,670	97	9,244,021	47,903,894	23,469	193	5790	8,196,757	349	42,422	1416
**Guangdong**	880,306	627	50,599,845	189,678,300	731,653	212	50,612,755	265,791,237	148,653	415	12,910	76,112,937	512	183,240	5896
**Guangxi**	245,019	254	19,747,045	44,884,943	202,089	85	19,752,230	94,521,577	42,930	169	5185	49,636,634	1156	292,976	9573
**Guizhou**	191,623	208	17,358,560	40,967,340	158,354	69	17,362,740	79,859,023	33,270	138	4180	38,891,683	1169	281,421	9303
**Hainan**	47,835	117	3,685,586	18,180,724	39,472	39	3,687,909	20,632,734	8363	78	2323	2,452,010	293	31,465	1056
**Hebei**	313,238	525	23,134,873	82,191,169	257,155	175	23,145,578	108,914,561	56,083	350	10,706	26,723,392	476	76,359	2496
**Heilongjiang**	74,939	81	4,698,470	14,254,947	61,710	27	4,700,130	23,247,845	13,229	54	1660	8,992,898	680	167,822	5418
**Henan**	377,667	428	35,682,912	73,521,845	311,441	143	35,691,677	151,441,346	66,227	285	8765	77,919,502	1177	273,435	8890
**Hubei**	196,493	138	16,273,006	30,573,339	162,430	46	16,275,857	68,728,885	34,063	92	2851	38,155,546	1120	415,738	13,384
**Hunan**	203,796	110	18,892,166	28,153,359	168,498	37	18,894,475	78,536,258	35,298	73	2308	50,382,899	1427	688,963	21,826
**Inner Mongolia**	99,927	170	6,256,911	26,762,166	82,146	57	6,260,354	33,616,615	17,781	113	3443	6,854,449	385	60,569	1991
**Jiangsu**	274,326	90	20,797,676	43,817,743	227,327	30	20,799,657	99,474,417	46,999	60	1981	55,656,674	1184	933,897	28,096
**Jiangxi**	164,847	400	14,402,696	54,468,030	135,480	134	14,410,676	69,129,350	29,366	266	7981	14,661,320	499	55,100	1837
**Jilin**	78,735	109	4,470,849	17,009,895	64,607	36	4,473,085	22,243,195	14,128	73	2236	5,233,300	370	72,170	2340
**Liaoning**	153,201	53	8,589,056	20,791,419	126,804	18	8,590,210	41,705,288	26,397	35	1155	20,913,869	792	600,666	18,112
**Ningxia**	38,912	85	3,030,263	11,647,699	32,066	28	3,031,948	15,597,964	6846	56	1685	3,950,266	577	70,181	2345
**Qinghai**	34,254	134	2,357,997	16,091,443	28,122	45	2,360,659	14,565,878	6132	90	2662	−1,525,565	Cost-saving	Cost-saving	Cost-saving
**Shaanxi**	173,995	370	13,478,596	52,623,337	143,698	124	13,485,983	69,447,588	30,297	246	7387	16,824,251	555	68,466	2277
**Shandong**	398,760	169	34,677,777	62,636,569	330,488	56	34,681,417	148,148,362	68,272	112	3640	85,511,793	1253	761,947	23,493
**Shanghai**	93,800	51	5,221,062	22,659,421	78,839	18	5,222,092	28,813,639	14,961	33	1030	6,154,218	411	188,800	5973
**Shanxi**	150,640	288	10,945,374	36,554,226	123,965	96	10,951,180	50,202,488	26,675	192	5806	13,648,262	512	71,164	2351
**Sichuan**	389,179	538	23,542,758	90,037,479	321,954	181	23,553,498	122,210,881	67,226	357	10,740	32,173,402	479	90,188	2996
**Tianjin**	54,471	50	3,142,640	15,078,365	45,095	17	3,143,665	18,205,665	9376	33	1025	3,127,299	334	94,028	3050
**Tibet**	29,084	138	1,544,930	15,401,124	23,999	46	1,547,595	11,402,574	5086	92	2665	−3,998,549	Cost-saving	Cost-saving	Cost-saving
**Xinjiang**	84,431	383	6,168,495	43,914,042	69,554	128	6,175,970	38,860,505	14,877	255	7475	−5,053,538	Cost-saving	Cost-saving	Cost-saving
**Yunnan**	322,268	781	17,854,524	107,348,497	266,083	262	17,869,929	107,238,381	56,184	519	15,405	−110,116	Cost-saving	Cost-saving	Cost-saving
**Zhejiang**	299,532	138	17,945,673	56,195,006	250,527	48	17,948,564	90,869,149	49,005	90	2892	34,674,143	708	383,243	11,992
**East**	2,441,338	1371	161,771,452	477,800,403	2,030,090	465	161,800,065	801,469,276	411,248	906	28,613	323,668,873	787	357,136	11,312
**Central**	1,831,523	2418	151,065,571	397,787,484	1,509,230	809	151,114,732	675,036,616	322,292	1609	49,161	277,249,132	860	172,287	5640
**West**	1,858,524	3445	129,205,607	513,907,864	1,534,414	1153	129,274,167	677,179,027	324,110	2291	68,561	163,271,164	504	71,253	2381
**National**	6,131,384	7234	442,042,629	1,389,495,750	5,073,734	2427	442,188,965	2,153,684,919	1,057,650	4807	146,335	764,189,169	723	158,976	5222

Rows and columns may not sum to the total due to rounding.

Spn: pneumococcal.

Nationally, including PCV13 in the NIP was estimated to cost US$ 2.0 billion in vaccine procurement, programmatic costs, and indirect costs (Table 2 and Webappendix 6: Table S2). However, vaccination costs would be partially mitigated by cost reductions of US$ 1.2 billion from averted treatment costs and increased lifetime productivity.

### Cost-effectiveness analysis

**Fig. 2:**
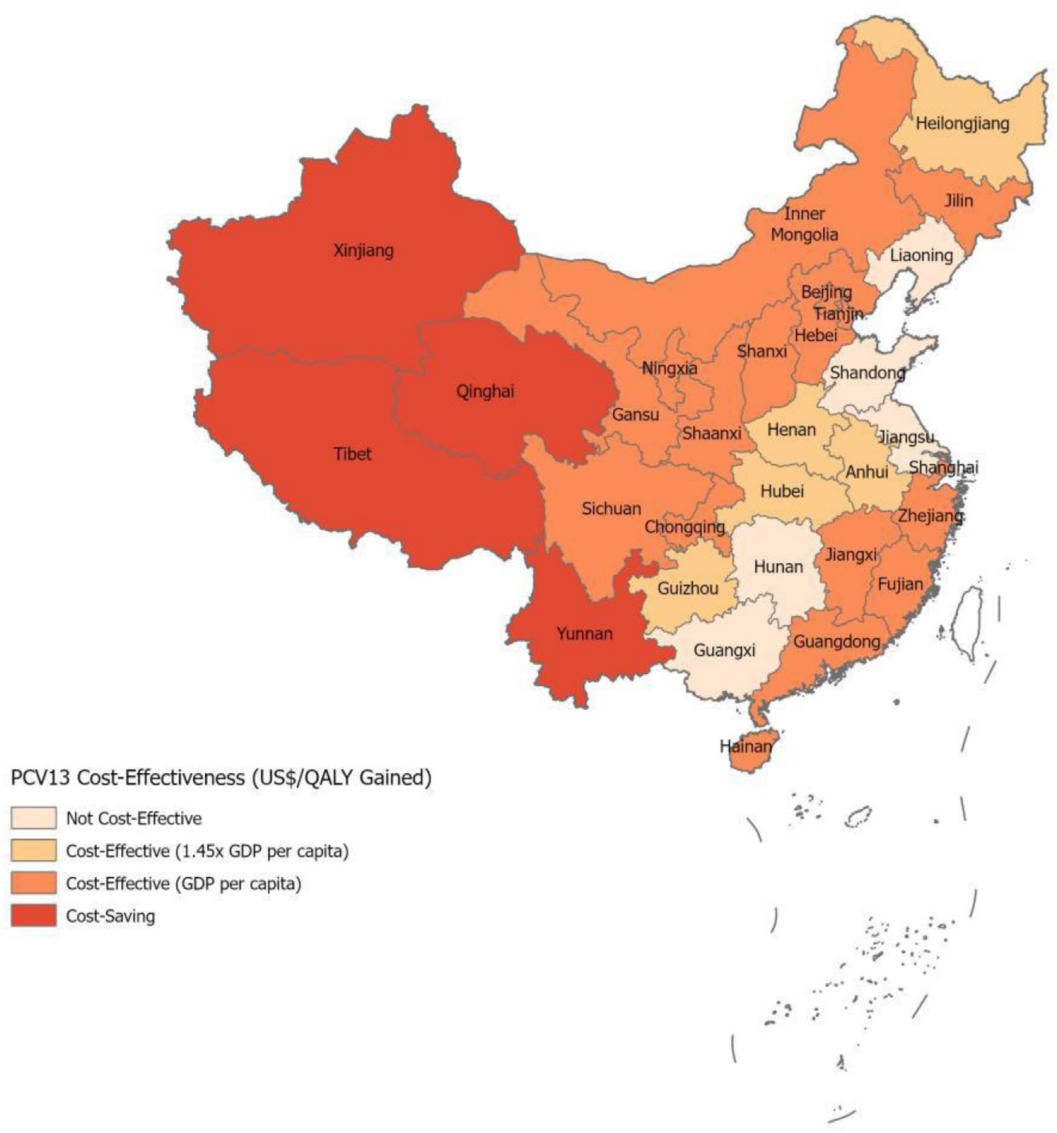
**Cost-effectiveness of PCV13 introduction in the national immunization program by province**. The map indicates provinces where PCV13 in the national immunization program is cost-effective compared to the *status quo* when the ICER (US$/QALY gained) is less than the provincial GDP per capita and the Cai et al. threshold of 1.45x the provincial GDP per capita.

The national ICERs per case averted, per death averted, and per QALY gained were US $723, US $158,976, and US $5222, respectively. The ICER per QALY gained was cost-effective compared to the national GDP per capita (US$ 10,274) in 2019 and the Cai et al. threshold for China (US$ 14,897). At the provincial level, adding PCV13 to the NIP was cost-effective in 21 and 26 of the 31 provinces when compared to the provincial GDP per capita and Cai et al. threshold,^43^ respectively (Fig. 2), and it was cost-saving in Qinghai, Tibet, Xinjiang and Yunnan provinces—all in the west region. Although PCV coverage in the private market was low nationally, the provinces where PCV vaccination was not cost-effective had lower disease burden and higher vaccine coverage in the private market compared to the other provinces. Conversely, the four provinces where PCV13 was cost-saving had high pneumococcal incidence and high CFRs while PCV coverage in the private market was approximately 0% (Webappendix 3: Table S1; Webappendix 5: Table S2).

### Sensitivity analysis

**Fig. 3:**
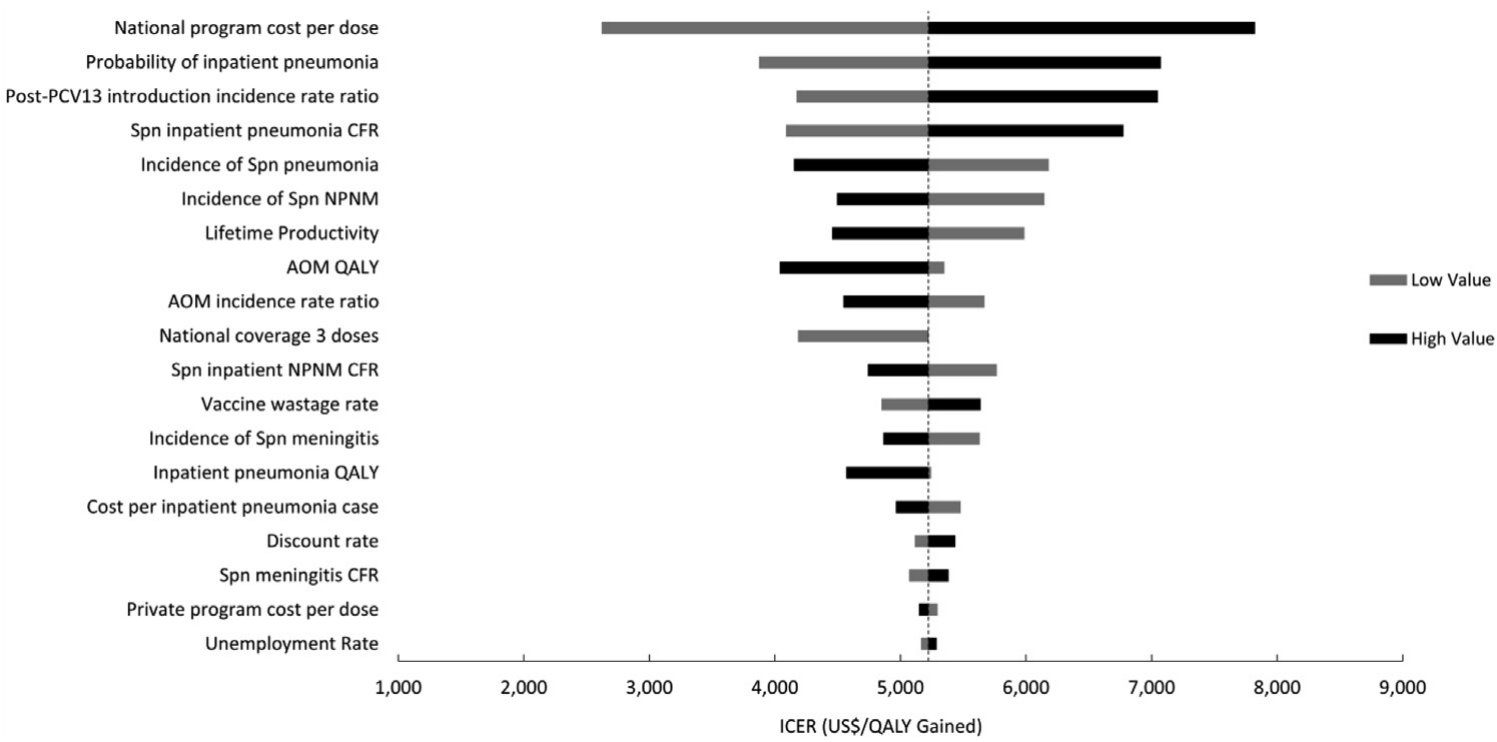
**Tornado diagram of one-way sensitivity analyses for the most influential model parameters on ICER (US$/QALY gained)**. When varying the parameters, the ICERs (US$/QALY gained) remained less than the national GDP per capita and the Cai et al. threshold of 1.45x the national GDP per capita.

**Fig. 4:**
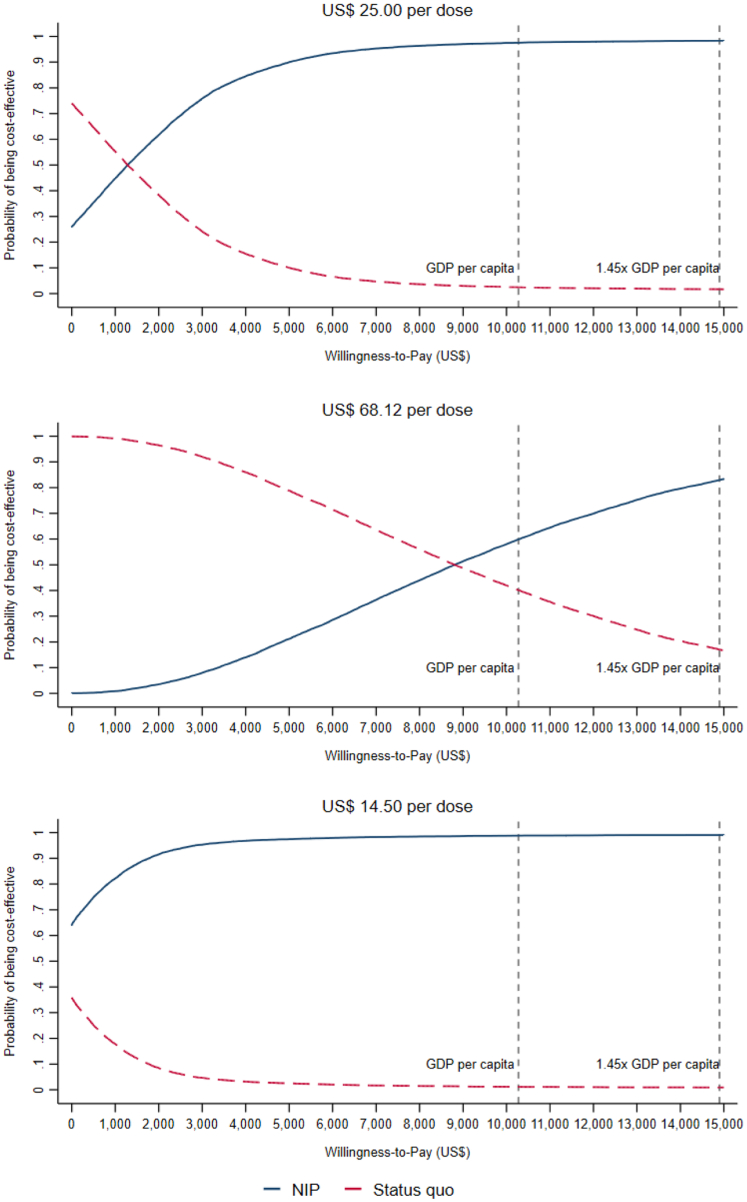
**Cost-effectiveness acceptability curves of the 3-dose national PCV13 program for the base case and different vaccine price scenarios**. Vertical lines represent the estimated threshold of 1.45x the 2019 national GDP per capita (US$ 14,897) by Cai et al.^43^ and the 2019 national GDP per capita (US$ 10,274). The probability that adding PCV13 into the National Immunization Program is cost-effective for the base case is 98% and increases to 99% when reducing the price of PCV13 to the PAHO price from the probabilistic sensitivity analysis (PSA). The probability decreases to 60% if the price remains at the private market rate.

The results of the deterministic sensitivity analyses were shown in a tornado diagram (Fig. 3), and the input variables most sensitive to changes in their value were price per dose in the NIP, disease burden estimates for pneumococcal pneumonia, and the post-PCV13 incidence rate ratio, while changing the discount rate from 0 to 10% did not show much influence on the results. Variations in these parameters within the specified ranges did not change the cost-effectiveness nationally compared to both the national GDP per capita threshold and the Cai et al. threshold. In the PSA, PCV13 in the NIP had a 98% probability of being cost-effective nationally compared to the national GDP per capita. The probability would decrease to 60% when raising the price of PCV13 to the current private market price, and increase to 99% when reducing the price to the PAHO price (Fig. 4). Provinces where PCV13 vaccination was not cost-effective in the base case would achieve reversed results if the NIP price was reduced. Adding 3-dose PCV13 to the NIP became cost-effective in all provinces when the NIP price was set at US$ 9 per dose (Webappendix 7: Table S2).

**Fig. 5:**
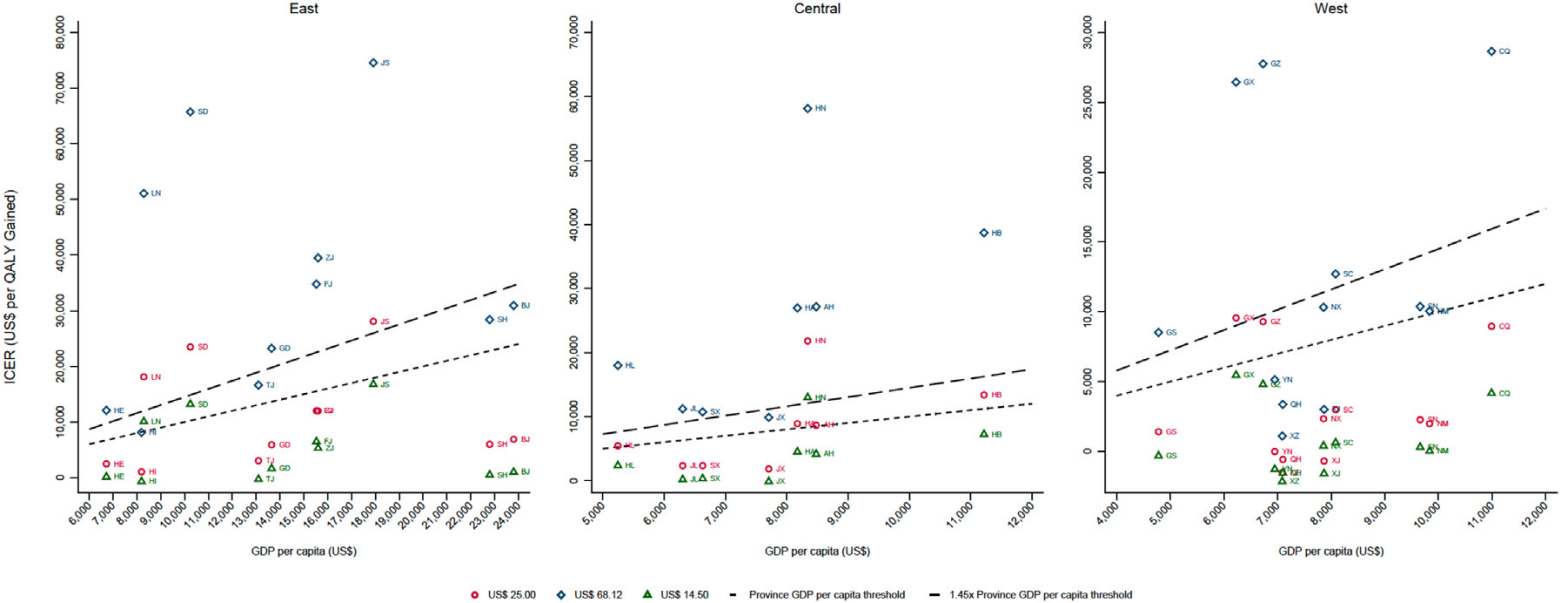
**Cost-effectiveness of 3-dose PCV13 introduction in the national immunization program by region and province for different vaccine price scenarios**. The graphs show ICERs of PCV13 vaccination in the NIP versus *status quo* in the private market by region and province versus the provincial GDP per capita. Different colours and shapes represent the base case and different vaccine price scenarios. The y-axis represents the cost-effectiveness estimate obtained from our model. The dashed lines represent the cost-effectiveness thresholds of the province GDP per capita and 1.45x the province GDP per capita. ICERs below the lines are cost-effective at that threshold. Credible intervals were omitted for clarity.

In scenarios under the 3-dose immunization schedule (Fig. 5 and Webappendix 7: Table S3), when the NIP vaccine price increased from US$ 25 (Upper bound recommended by UNICEF for MICs) to US$ 68.12 (private market price), the national ICER per QALY gained increased from US$ 5.222 to US$ 18,622, at which point PCV13 in the NIP was cost-effective in only 5 provinces. Conversely, if the NIP vaccine price decreases from US$ 25 to US$ 14.5 (PAHO price), the national ICER would decrease to US$ 1959, at which point PCV13 was cost-effective in 28 provinces. If we further separated provinces by region (Fig. 5), the west region had higher cost-effectiveness of PCV13 introduction compared to the central and east regions in different scenarios. As for vaccination schedules, a 4-dose schedule (3 + 1) would decrease the cost-effectiveness of PCV13 for all prices, but it would remain cost-effective for the base case (US$ 25) and PAHO price (US$ 14.5) (Webappendix 7: Table S4). Scenario analyses on IPD IRR and NIP coverage rates also indicated robust cost-effectiveness results at the nation level (Webappendix 7: Tables S5 and S6).

## Discussion

This is the first study to assess the cost-effectiveness of introducing PCV13 into the NIP compared to the *status quo* in the private market for all 31 provinces in China. For a single hypothetical birth cohort, the introduction of PCV13 into the NIP could save approximately 4807 lives (66% reduction) and avert over 1,057,650 cases (17% reduction) of pneumococcal disease. There was a US$ 1^.^2 billion reduction in costs from averted treatment expenses and increased lifetime productivity. From a societal perspective, PCV13 in the NIP was cost-effective nationally compared to the 2019 GDP per capita with an ICER per QALY gained of US$ 5222. The results offered a compelling case for the Chinese government to support a PCV13 program in the NIP for young children as recommended by the WHO.

The access and coverage of PCV13 in the private market remained low and varied substantially by province in China. The national 3-dose vaccination rate of PCV13 in 2017 was only 1.3%, far lower than the global average of 49%.^44^ The coverage also ranged from over 7% in higher socioeconomically developed provinces like Beijing and Shanghai to nearly 0% in less socioeconomically developed provinces in the west region, like Tibet and Qinghai. The present study indicated that PCV13 in the NIP was cost-effective in 21 of 31 Chinese provinces with the west region getting the largest benefit. Expanded PCV vaccination was most cost-effective in provinces with higher pneumococcal disease burden and/or nearly 0% coverage in the private market. In less socioeconomically developed provinces, mostly in the west region, pneumococcal disease burden remained high while access to vaccines in the private market was limited. In Qinghai, Tibet, Xinjiang and Yunnan, all provinces with low PCV coverage in the private market and high mortality, introducing PCV13 into these provinces was cost-saving. Adding PCV13 into China's NIP would reduce the disease burden especially in the west region and also promote health equity by expanding access to the vaccine for children throughout China.

As the first study comparing national vaccination with the *status quo* in the private market in China, our findings were consistent with other studies comparing national vaccination to no vaccination, where the inclusion of compulsory childhood PCV vaccination in China also demonstrated substantial reductions in pneumococcal disease burden and associated costs.^45^, ^46^, ^47^, ^48^ However, the cost-effectiveness results of these studies varied by vaccine product choice, threshold setting, vaccine price, indirect effects, and differences in incidence estimates. Some studies indicated that a PCV13 program in China would be cost-effective,^45^, ^46^, ^47^ while one study suggested the opposite.^48^

The sensitivity analyses also demonstrated the robustness of the base case results, with vaccine price, pneumonia disease burden, and post-PCV incidence rate ratios being the main drivers of cost-effectiveness. Past experiences indicated that vaccine prices could reduce substantially when PCV13 could be added to the NIP after negotiations between the Chinese government and vaccine manufacturers because of a large volume purchased annually. Moreover, the newly-developed domestic PCV products in China also ensured the capacity for introducing and supporting pneumococcal vaccination in the NIP in the future. By 2019, the Gavi, The Vaccine Alliance provided immunizations for children across 60 lower-income countries against pneumococcal disease, and middle-income countries like South Africa and Brazil had added PCVs into their NIP.^49^ Because China was not Gavi-eligible, we assumed the base-case NIP PCV13 vaccine price was the upper bound price recommended by UNICEF for MICs, which was lower than the private market price but higher than the PAHO price. If we accounted for variability in vaccine price, PCV13, which was nationally cost-effective in the base case analysis, would not be cost-effective when the NIP vaccine price remained the same as the private market price (2.7 times the maximum price of UNICEF), but it would be more cost-effective if the NIP price was reduced to the PAHO price. The scenario sensitivity analysis also indicated that 3-dose PCV13 schedule was more cost-effective than the 4-dose schedule, but the choice of preferred immunization schedules needed further consideration in addition to economic evidence.

China has a large population with substantial subnational socioeconomic, cultural, and geographic differences. To inform China's immunization policies, this study also conducted cost-effectiveness analysis at the provincial level to examine regional and provincial disparities. By comparison, the less-developed west region had a higher pneumococcal disease burden and lower PCV coverage in the private sector compared to the east and central regions, despite it only accounting for 29% of the live births in 2019. Therefore, the west region will achieve higher cost-effectiveness if PCV13 is included in the NIP. Provincial governments in China are empowered to include specific vaccines into local immunization programmes, and our provincial estimates provide new evidence to support the introduction of PCV13 in different provinces. The less-developed west region is in more urgent need of PCV to reduce pneumococcal disease burden among young children. It was also noted that PCV13 introduction was not cost-effective in several provinces even under the higher threshold, including Hunan, Jiangsu, Liaoning, Guangxi and Shandong, due to their lower estimated pneumococcal disease incidence or CFR. However as shown in the sensitivity analysis, those provinces could also achieve cost-effectiveness by means of centralized procurement in large quantities to reduce vaccine prices. Regions with higher economic development levels might consider taking the lead in incorporating PCV13 into their local childhood immunization programmes after centralized procurement. Introducing PCV13 in the NIP or in high-burden provinces should be a key strategy to meet the Sustainable Development Goal child survival targets by 2030 and accelerate the elimination of pneumococcal disease globally.

There were also a number of limitations in this study. First, a lack of reliable provincial-level data on access to care and deaths occurring outside health facilities might influence the results. Given the recorded high national access to care in China, we assumed all pneumococcal cases sought care at health facilities and all deaths occurred in hospitals. Reductions in care-seeking, particularly among AOM cases, would reduce cost savings and increase ICER estimates, while deaths occurring outside health facilities could result in underestimating the ICERs. Besides, the incidence of pneumococcal disease in adults would also decrease when PCV13 could be widely used among children. This study did not involve the impact of childhood PCV13 vaccination on the pneumococcal disease burden among adults, resulting in a conservative estimate for the effects of a universal PCV13 vaccination program. While it would be more precise to have week-by-week or month-by-month models to differentiate the first few months before and after the vaccination of PCV doses, data on the disease burden and probability of death (natural or due to pneumococcus) were not available at this level of granularity, and we only included children surviving past the neonatal period to more accurately reflect the cohort of children that were eligible for vaccination. For the same reason, we did not account for the transition from IPD to healthy state to non-Spn death within a one-year cycle. Recovery time from severe IPD could take several months, and data on the change in mortality risk during the period following recovery were not available. However, pneumococcal infection increased the risk of dying from comorbidities or secondary infections, and not including these in the model could underestimate the impact of PCV13 on deaths averted and underestimate the cost-effectiveness. Last, we constructed the model using the latest available estimates from reasonably nationally representative sources, but some data might lack representativeness. Utilities for health states were unavailable in China, and utilities derived from foreign studies might be subject to systematic differences in how utility was perceived among the populations of these countries. Using CHIRA data might overestimate the costs of pneumococcal disease because a majority of the insured population resided in urban areas, and thus would underestimate the ICERs in provinces with large rural populations. At the same time, some costs difficult to be accounted for were not considered in this study, such as the direct non-medical costs and caregiver productivity losses for meningitis sequelae cases, and the costs for potential sequelae of pneumonia, NPNM and AOM cases, which might underestimate the cost-effectiveness. Despite such limitations, the sensitivity analysis showed that variations in health utilities or treatment costs were not likely to change the cost-effectiveness in individual provinces, so the lack of representativeness in these model estimates would have minimal impact on the cost-effectiveness results.

### Conclusion

This study provided evidence to support the introduction of PCV13 into China's NIP at a lower price as it was cost-effective nationally. PCV13 was cost-effective in the majority of provinces in China, and even cost-saving in four west provinces. The provincial results supported subnational introduction of PCV13 in the absence of a national policy decision on PCV13, and priority should be given to less socioeconomically developed provinces like Tibet, Xinjiang, Qinghai and Yunnan due to their higher pneumococcal disease burden and substantial benefits gained from including PCV13 in the NIP. Efforts to improve the affordability of PCV13 after negotiations will benefit public health in a cost-effective manner, and the choice of vaccination schedule needs further consideration.

## Contributors

XL, CG and DW did the analyses for vaccine cost-effectiveness and wrote the first draft of the manuscript. MDK, BW, ZY, and HF designed the project and oversaw the analysis and manuscript writing. XL, HZ, TX, RJ and DW prepared provincial vaccine coverage and population data in China. HF supervised the entire project and acquired funding. All coauthors provided feedback during the design and interpretation of the project. They also contributed to revisions of the manuscript. All authors read and approved the final manuscript. XL, CG and DW contributed equally as the co-first authors. ZY, BW, and HF contributed equally as the senior authors.

## Data sharing statement

XL and HF had full access to all the data in the study and take responsibility for the integrity of the data and the accuracy of the data analysis. Partial data are available on appendix and readers can also contact HF (one of corresponding author) for other data.

## Editor note

The Lancet Group takes a neutral position with respect to territorial claims in published maps and institutional affiliations.

## Declaration of interests

HF reports grants from the 10.13039/100000865Bill & Melinda Gates Foundation and 10.13039/100014588Sanofi Pasteur. BW reports grants from the 10.13039/100000865Bill & Melinda Gates Foundation. MDK reports grants from the 10.13039/100000865Bill & Melinda Gates Foundation, the 10.13039/100004423World Health Organization, 10.13039/100004334Merck, 10.13039/100004319Pfizer and Gavi Alliance, and personal fees from Merck. CG reports grants from the 10.13039/100000865Bill & Melinda Gates Foundation and 10.13039/100004319Pfizer, and personal fees from 10.13039/100004334Merck. All other authors declare no competing interests.

## Appendix ASupplementary data

Supplementary appendix

Caption for supplementary material
