# Supplementary material for: Estimating national, regional and provincial cost-effectiveness of introducing childhood 13-valent pneumococcal conjugate vaccination in China: a modelling analysis
Source: Lancet Reg Health West Pac. 2022 Dec 19;32:100666. doi: 10.1016/j.lanwpc.2022.100666 (PMC9918781; doi:10.1016/j.lanwpc.2022.100666)
Supplement: Supplementary appendix [file mmc1.pdf]

Supplementary appendix for

Estimating national, regional and provincial cost-effectiveness of introducing childhood 13-valent pneumococcal conjugate vaccination in China: a modelling analysis

Authors: Xiaozhen Lai,[1,2*] Cristina Garcia,[3,4*] Dan Wu,[5*] Maria Deloria Knoll,[3,4] Haijun Zhang,[1,2] Tingting Xu,[6] Rize Jing,[7] Zundong Yin,[5†] Brian Wahl,[3,4†] Hai Fang[1,8,9†]

[1] China Center for Health Development Studies, Peking University, Beijing, China

[2] Department of Health Policy and Management, School of Public Health, Peking University, Beijing, China

[3] Department of International Health, Johns Hopkins Bloomberg School of Public Health, Baltimore, USA

[4] International Vaccine Access Center, Johns Hopkins Bloomberg School of Public Health, Baltimore, USA

[5] National Immunization Program, Chinese Center for Disease Control and Prevention, Beijing, China

[6] Department of Health Management and Policy, School of Public Health, Capital Medical University, Beijing, China

[7] School of Public Administration and Policy, Renmin University of China, Beijing, China

[8] Peking University Health Science Center, Chinese Center for Disease Control and Prevention Joint Research Center for Vaccine Economics, Beijing, China

[9] Institute for Global Health and Development, Peking University, Beijing, China

[*] contributed equally

[†] contributed equally

Correspondence:

Zundong Yin, Chinese Center for Disease Control and Prevention, Beijing, China, E-mail: yinzd@chinacdc.cn

Brian Wahl, International Vaccine Access Center, Johns Hopkins Bloomberg School of Public Health, Baltimore, USA, Email: bwahl@jhu.edu

Hai Fang, China Center for Health Development Studies, Peking University, Beijing, China, E-mail: hfang@hsc.pku.edu.cn

Funding: Bill & Melinda Gates Foundation.

Keywords: *Streptococcus pneumoniae*, Pneumococcal conjugate vaccination, China, Child health, Health economics, Economic analysis

补充附录

估计中国将儿童 13 价肺炎球菌结合疫苗纳入免疫规划的国家、区域和省级成本效益：一项模型分析

作者： 来晓真,[1,2*] Cristina Garcia,[3,4*] 吴丹,[5*] Maria Deloria Knoll,[3,4] 张海军,[1,2] 徐婷婷,[6], 景日泽,[7] 尹遵栋,[5†] Brian Wahl,[3,4†] 方海 [1,8,9†]

[1] 北京大学中国卫生发展研究中心，北京，中国

[2] 北京大学公共卫生学院卫生政策与管理系，北京，中国

[3] 约翰斯·霍普金斯大学布隆伯格公共卫生学院国际卫生系，巴尔的摩，美国

[4] 约翰斯·霍普金斯大学布隆伯格公共卫生学院国际疫苗获取中心，巴尔的摩，美国

[5] 中国疾病预防控制中心免疫规划中心，北京，中国

[6] 首都医科大学公共卫生学院卫生管理与政策系，北京，中国

[7] 中国人民大学公共管理学院，北京，中国

[8] 北京大学医学部-中国疾病预防控制中心疫苗经济学联合研究中心，北京，中国

[9] 北京大学全球健康发展研究院，北京，中国

[*] 贡献等同

[†] 贡献等同

通讯作者：

尹遵栋，中国疾病预防控制中心免疫规划中心，北京，中国，电子邮箱：yinzd@chinacdc.cn

Brian Wahl，约翰斯·霍普金斯大学布隆伯格公共卫生学院国际疫苗获取中心，巴尔的摩，美国，电子邮箱：bwahl@jhu.edu

方海，北京大学中国卫生发展研究中心，北京，中国，电子邮箱：hfang@hsc.pku.edu.cn

中文摘要

背景

在中国，尽管 13 价肺炎球菌结合疫苗（PCV13）在私人市场有售，但尚未将其纳入国家免疫规划（NIP）。本研究旨在评估在国家和省级层面将 PCV13 纳入中国 NIP 的成本效益。

方法

我们使用决策树马尔可夫模型，从全社会角度估计与私人市场现状相比，将 3 剂次 PCV13 纳入中国 NIP 中的成本效益。该模型假定在疫苗纳入 5 年后的一个出生队列。治疗成本和免疫规划成本根据中国疾病预防控制中心（CDC）和国家保险数据库计算得出。疾病负担数据、相对发病率指数和其他模型参数来自已发表和灰色文献。在省级层面估计了避免的病例和死亡、获得的质量调整生命年（QALY）和增量成本效益比（ICER）。研究也开展了单因素、情景和概率敏感性分析以探索模型的不确定性。

结果

在 2019 年出生队列的前 5 年中, 从国家层面将 PCV13 纳入 NIP 中预计可预防约 4 807 例肺炎球菌疾病死亡(降低 66%)和 1 057 650 例肺炎球菌病例(降低 17%)。在 NIP 价格假设每剂 25 美元的基础情境下，将 PCV13 纳入 NIP 在全国范围内具有成本效益，避免每 QALY 损失的 ICER 为 5 222 美元，在 31 个省级地区中，17 个具有成本效益，4 个成本节约。单因素和情景敏感性分析表明，改变不同模型参数后结果仍稳健，概率敏感性分析表明 PCV13 在全国范围内具有成本效益的可能性为 98%。

解释

本研究结果突出了将 PCV13 纳入中国 NIP 中的成本效益。省级结果支持各地区纳入 PCV13，且应优先考虑社会经济欠发达省份。由于接种费用是影响最大的模型参数，通过集中采购努力提高 PCV 的可负担性将改善成本效益，为公共卫生带来益处。

基金项目：比尔及梅琳达·盖茨基金会

关键词：肺炎链球菌，肺炎球菌结合疫苗，中国，儿童健康，卫生经济，经济学评价

Editor note: This translation in Chinese was submitted by the authors and we reproduce it as supplied. It has not been peer reviewed. Our editorial processes have only been applied to the original abstract in English, which should serve as reference for this manuscript.

Webappendix List

- Webappendix 1: CHEERS 2022 Checklist
- Webappendix 2: Data sources for demographic model parameters in economic model
- Webappendix 3: Data sources and methods for estimating pneumococcal epidemiological parameters by province
- Webappendix 4: Methods for estimating direct and indirect costs of pneumococcal diseases by province
- Webappendix 5: Data sources and methods for estimating PCV coverage for each strategy and the cost of immunization delivery per dose by province
- Webappendix 6: Provincial disease burden and economic supplemental results
- Webappendix 7: Incremental cost-effectiveness ratios of including PCV13 in the NIP for the base case and sensitivity analysis

Webappendix 1. CHEERS 2022 Checklist

Topic	No.	Item	Location where item is reported
**Title**			
	1	Identify the study as an economic evaluation and specify the interventions being compared.	Title, Page 1
**Abstract**			
	2	Provide a structured summary that highlights context, key methods, results, and alternative analyses.	Summary, Page 3
**Introduction**			
**Background and objectives**	3	Give the context for the study, the study question, and its practical relevance for decision making in policy or practice.	Introduction, Page 5
**Methods**			
**Health economic analysis plan**	4	Indicate whether a health economic analysis plan was developed and where available.	Methods, First and second paragraphs
**Study population**	5	Describe characteristics of the study population (such as age range, demographics, socioeconomic, or clinical characteristics).	Methods, First paragraph
**Setting and location**	6	Provide relevant contextual information that may influence findings.	Methods, First paragraph
**Comparators**	7	Describe the interventions or strategies being compared and why chosen.	Methods, First paragraph
**Perspective**	8	State the perspective(s) adopted by the study and why chosen.	Methods, Second paragraph
**Time horizon**	9	State the time horizon for the study and why appropriate.	Methods, Second and ninth paragraphs
**Discount rate**	10	Report the discount rate(s) and reason chosen.	Methods, Second paragraph
**Selection of outcomes**	11	Describe what outcomes were used as the measure(s) of benefit(s) and harm(s).	Methods, Third and ninth paragraphs
**Measurement of outcomes**	12	Describe how outcomes used to capture benefit(s) and harm(s) were measured.	Methods, Third and ninth paragraphs
**Valuation of outcomes**	13	Describe the population and methods used to measure and value outcomes.	Methods, Third and ninth paragraphs
**Measurement and valuation of resources and costs**	14	Describe how costs were valued.	Methods, Fourth, fifth and eighth paragraphs

Topic	No.	Item	Location where item is reported
**Currency, price date, and conversion**	15	Report the dates of the estimated resource quantities and unit costs, plus the currency and year of conversion.	Methods, Second paragraph
**Rationale and description of model**	16	If modelling is used, describe in detail and why used. Report if the model is publicly available and where it can be accessed.	Methods, Third and ninth paragraphs and Appendix
**Analytics and assumptions**	17	Describe any methods for analysing or statistically transforming data, any extrapolation methods, and approaches for validating any model used.	Methods, Third paragraph and Appendix
**Characterising heterogeneity**	18	Describe any methods used for estimating how the results of the study vary for subgroups.	Methods, Third to eighth paragraphs (provincial-level data)
**Characterising distributional effects**	19	Describe how impacts are distributed across different individuals or adjustments made to reflect priority populations.	Methods, Third to eighth paragraphs (provincial-level data)
**Characterising uncertainty**	20	Describe methods to characterise any sources of uncertainty in the analysis.	Methods, Tenth and eleventh paragraphs
**Approach to engagement with patients and others affected by the study**	21	Describe any approaches to engage patients or service recipients, the general public, communities, or stakeholders (such as clinicians or payers) in the design of the study.	Not applicable
**Results**			
**Study parameters**	22	Report all analytic inputs (such as values, ranges, references) including uncertainty or distributional assumptions.	Table 1 and Appendix
**Summary of main results**	23	Report the mean values for the main categories of costs and outcomes of interest and summarise them in the most appropriate overall measure.	Results, First to third paragraphs
**Effect of uncertainty**	24	Describe how uncertainty about analytic judgments, inputs, or projections affect findings. Report the effect of choice of discount rate and time horizon, if applicable.	Results, Fourth and fifth paragraphs
**Effect of engagement with patients and others affected by the study**	25	Report on any difference patient/service recipient, general public, community, or stakeholder involvement made to the approach or findings of the study	Not applicable

Topic	No.	Item	Location where item is reported
**Discussion**			
**Study findings, limitations, generalisability, and current knowledge**	26	Report key findings, limitations, ethical or equity considerations not captured, and how these could affect patients, policy, or practice.	Discussion
**Other relevant information**			
**Source of funding**	27	Describe how the study was funded and any role of the funder in the identification, design, conduct, and reporting of the analysis	Methods, Last paragraph
**Conflicts of interest**	28	Report authors conflicts of interest according to journal or International Committee of Medical Journal Editors requirements.	End of manuscript

Webappendix 2. Data sources for demographic model parameters in economic model

Province-level data for the 2019 live birth cohort were obtained from the 2020 China Health Statistical Yearbook.[1] All-cause mortality rates for neonates and under-five year-olds were obtained from a 2016 cause of death systematic review[2] and the 2019 Global Burden of Disease (GBD) study in China, respectively.[3] Song et al. 2016 identified high-quality community-based longitudinal cause of death studies in under-five children in China and modelled all-cause mortality by province for 2015.[2] For the economic model, we assumed the mortality rates remained stable from 2015 to 2019. The provincial birth cohorts and mortality rates used in the economic model are described in Table 1.

Table 1. Model live birth cohort and mortality rate parameters by province

Province	Abbr.	GDP Per Capita in RMB (US$)	2019 Live Birth Cohort	Neonatal mortality rate (per 1,000 live births)	Under five mortality rate (per 1,000 live births)
Anhui	AH	58,496 (8,478)	618,040	5.5	12.0
Beijing	BJ	164,220 (23,800)	218,006	1.9	5.0
Chongqing	CQ	75,828 (10,990)	282,543	6.0	13.0
Fujian	FJ	107,139 (15,527)	461,679	4.0	7.0
Gansu	GS	32,995 (4,782)	304,092	10.8	20.0
Guangdong	GD	94,172 (13,648)	1,653,153	2.7	7.0
Guangxi	GX	42,964 (6,227)	646,459	5.3	12.0
Guizhou	GZ	46,433 (6,729)	569,987	9.0	18.0
Hainan	HI	56,507 (8,189)	120,932	7.5	16.0
Hebei	HE	46,348 (6,717)	757,878	5.9	13.0
Heilongjiang	HL	36,183 (5,244)	153,837	5.4	12.0
Henan	HA	56,388 (8,172)	1,168,310	5.7	13.0
Hubei	HB	77,387 (11,215)	532,659	5.3	12.0
Hunan	HN	57,540 (8,339)	617,999	4.7	9.0
Inner Mongolia	NM	67,852 (9,834)	205,132	6.6	13.0
Jiangsu	JS	123,607 (17,914)	679,171	2.5	7.0
Jiangxi	JX	53,164 (7,705)	472,492	7.3	16.0
Jilin	JL	43,475 (6,301)	146,201	3.5	9.0

Liaoning	LN	57,191 (8,289)	280,738	3.6	7.0
Ningxia	NX	54,217 (7,858)	99,448	7.9	16.0
Qinghai	QH	48,981 (7,099)	77,552	9.2	21.0
Shaanxi	SN	66,649 (9,659)	442,011	6.9	15.0
Shandong	SD	70,653 (10,240)	1,133,927	4.2	10.0
Shanghai	SH	157,279 (22,794)	170,577	2.8	7.0
Shanxi	SX	45,724 (6,627)	358,159	4.4	12.0
Sichuan	SC	55,774 (8,083)	771,667	6.6	15.0
Tianjin	TJ	90,371 (13,097)	102,720	3.2	8.0
Tibet	XZ	48,902 (7,087)	51,291	16.5	36.0
Xinjiang	XJ	54,280 (7,867)	203,758	13.2	28.0
Yunnan	YN	47,944 (6,948)	586,497	8.4	17.0
Zhejiang	ZJ	107,624 (15,598)	586,425	3.3	7.0

REFERENCES

1. National Health Commission of China. China Health Statistical Yearbook 2020. Beijing: Chinese Academy of Medical Sciences & Peking Union Medical College Press; 2020.

2. Song P, Theodoratou E, Li X, Liu L, Chu Y, Black RE, et al. Causes of death in children younger than five years in China in 2015: an updated analysis. *J Glob Health* 2016; **6**: 020802.

3. Zhou M, Wang H, Zeng X, Yin P, Zhu J, Chen W, et al. Mortality, morbidity, and risk factors in China and its provinces, 1990-2017: a systematic analysis for the Global Burden of Disease Study 2017. *Lancet* 2019; **394**: 1145–58.

Webappendix 3. Data sources and methods for estimating pneumococcal epidemiological parameters by province

The Markov model considered four clinical syndromes for pneumococcus: pneumonia, meningitis, invasive non-pneumonia, non-meningitis disease (NPNM), and acute otitis media (AOM). The provincial-level age-specific probabilities of IPD cases and deaths for children aged 1-59 months, including pneumococcal severe and non-severe pneumonia, meningitis and NPNM, were estimated from a separate disease burden model [1]. The case definitions and methods used in the disease burden model were previously described [2]. In the disease burden model, provincial-level pathogen-specific pneumonia and meningitis disease burden was reported from 2010 to 2017, and we further predicted provincial-level estimates in 2019 by applying the predicted proportions of pneumonia and meningitis cases and deaths for children in different age groups (neonatal, infant, under-five) in 2019 to the national-level estimates of all-cause pneumonia and meningitis disease burden data obtained from GBD IHME [1]. Pneumococcal morbidity and mortality estimates were prepared assuming no vaccine use. We then adjusted these estimates to account for vaccine use in the private sector and national sector as described in Webappendix 5. Detailed introduction to the disease burden model can be found as follows and accessed in a previously published study [1].

Pathogen-specific pneumonia model

Pathogen-specific pneumonia deaths and cases were prepared by applying estimates of the proportion of pneumonia deaths and cases attributable to each pathogen to all-cause pneumonia mortality and morbidity estimates (Figure 1, A. Pneumonia). The latter were obtained from annual modelled provincial-level estimates for children aged 1–59 months in China for 2010 to 2017 prepared by Global Burden of Disease, Injuries, and Risk Factors Study (GBD) [3]. We used values of PCV efficacy and effectiveness against WHO-defined clinical non-severe (outpatient) and severe (inpatient) pneumonia to estimate the contributions of each pathogen to these case definitions. The pneumococcal attributable fraction for pneumonia mortality was estimated using efficacy against radiograph-confirmed, primary endpoint pneumonia from vaccine clinical trials.[2] Efficacy against clinical severe pneumonia was used to estimate clinical severe pneumococcal pneumonia morbidity. Provincial pneumococcal pneumonia CFR values were estimated by dividing pneumococcal pneumonia mortality by clinical severe pneumococcal pneumonia cases.

Pathogen-specific meningitis model

For meningitis, we used estimates of the proportion of meningitis cases attributable to common bacterial pathogens and estimates of relative case fatality of these pathogens to estimate the proportion of meningitis deaths due to pneumococcus, as described in a previous publication.[2] For each province, we prepared summary estimates of each parameter by meta-analysing data from Asia and other epidemiologically relevant settings identified through a literature search. We stratified global data for pathogen-specific meningitis CFR by child mortality setting (i.e., <30 deaths per 1 000 livebirths, 30 to <75 deaths, and ⩾75 deaths), and applied them to each province in China based on their child mortality settings. We then applied the proportion of meningitis deaths caused by pneumococcus to modelled provincial all-cause deaths of meningitis aged 1–59 months from 2010 to 2017 prepared by GBD in China to estimate pathogen-specific meningitis deaths for each province, which were divided by pathogen-specific meningitis CFR estimates to derive pneumococcal meningitis morbidity estimates (Figure 1, B. Meningitis).

Pathogen-specific invasive non-pneumonia, non-meningitis model

Pathogen-specific morbidity from NPNM invasive syndromes (e.g., sepsis) were estimated by applying to the meningitis case estimates the pathogen-specific NPNM to meningitis case ratios, obtained from meta-estimates of published studies globally, stratified by high or very high (i.e., >75 deaths per 1 000 live births) and low or medium (i.e., <75 deaths per 1 000 live births) all-cause child mortality, as previously described.[2] NPNM deaths were estimated by multiplying country-specific NPNM cases (severe cases for pneumococcus) by country-specific NPNM CFR. The latter was derived by estimating a mortality setting-specific ratio of pathogen-specific NPNM CFR and pathogen-specific meningitis CFR, and applying that to the province-specific pneumococcal meningitis CFR (Figure 1, C. NPNM).

In the present study, global pneumococcal meningitis and pneumococcal NPNM CFR values stratified by all-cause child mortality strata were used at the provincial level.[2] All provinces except Tibet and Xinjiang were in the same all-cause child mortality stratum, and the same CFR values for pneumococcal meningitis (0.125; UR 0.058-0.271) and pneumococcal NPNM (0.098; UR 0.045-0.213) were applied. The CFR values for pneumococcal meningitis and pneumococcal NPNM for Tibet were

0.279 (UR 0.094-0.834) and 0.219 (UR 0.073-0.653), respectively. The CFR values for pneumococcal meningitis and pneumococcal NPNM for Xinjiang were 0.193 (UR 0.089-0.419) and 0.151 (UR 0.070-0.328), respectively.

Figure 1: Pathogen-specific pneumonia, meningitis, and NPNM morbidity and mortality conceptual models.

NPNM=non-pneumonia, non-meningitis. CFR=case-fatality ratio.

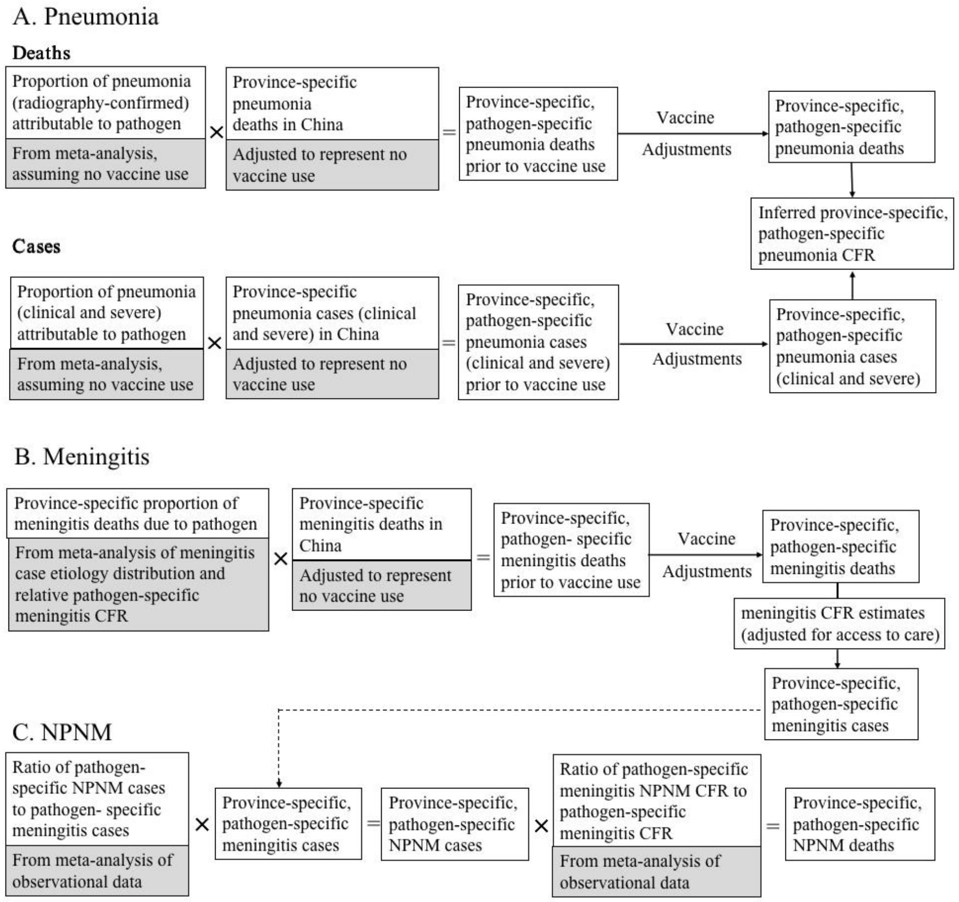

For AOM, we used national estimates of otitis media incidence obtained from the GBD 2019 results, to which the age-specific fraction of AOM was applied.[4] We

also conducted a review of currently available studies reporting AOM incidence in one or more provinces in China, and published data on the provincial-level AOM disease burden was limited. No studies with estimates for all provinces were available. To adjust the national estimates by province, we assumed the national to province ratio of AOM was similar to that of pneumonia and applied the provincial-level ratio of pneumococcal pneumonia to estimate provincial-level AOM incidence.

The incidence estimates used in the economic model assumed no vaccine effect (i.e., no PCV vaccination publicly or privately) because the vaccine effect was applied in the Markov model based on the vaccination strategy and corresponding coverage and costs. Because access to care was high in all provinces,[5] we assumed all severe disease (severe pneumonia, meningitis, and NPNM) resulted in hospitalization, and all deaths occurred in the hospital.

Table 1. Pneumococcal disease burden among children 1-59 months by province.

Province	Spn pneumonia incidence per 100,000 (UR)	Spn meningitis incidence per 100,000 (UR)	Spn NPNM incidence per 100,000 (UR)	Inpatient Spn pneumonia CFR (UR)	All-cause AOM incidence per 100,000 (UR)
Anhui	552 (476-656)	5 (3-7)	20 (12-27)	0.024 (0.015-0.034)	6663 (6621-6817)
Beijing	874 (754-1039)	4 (2-5)	16 (10-22)	0.013 (0.008-0.018)	10554 (10488-10799)
Chongqing	626 (540-744)	4 (3-6)	18 (11-24)	0.020 (0.012-0.027)	7560 (7513-7735)
Fujian	540 (466-642)	4 (3-6)	17 (11-23)	0.021 (0.013-0.030)	6523 (6483-6675)
Gansu	670 (578-796)	15 (9-20)	61 (38-82)	0.052 (0.033-0.073)	8089 (8038-8276)
Guangdong	804 (694-956)	6 (3-7)	23 (14-31)	0.019 (0.012-0.026)	9714 (9653-9939)
Guangxi	575 (496-684)	4 (3-6)	18 (11-25)	0.026 (0.016-0.037)	6949 (6905-7110)
Guizhou	516 (446-614)	3 (2-4)	13 (8-17)	0.032 (0.02-0.044)	6239 (6200-6384)
Hainan	605 (522-719)	10 (6-14)	42 (26-57)	0.066 (0.041-0.092)	7307 (7261-7477)
Hebei	625 (539-743)	12 (8-16)	50 (31-68)	0.030 (0.019-0.042)	7547 (7499-7722)
Heilongjiang	739 (638-879)	9 (6-12)	38 (23-51)	0.020 (0.012-0.028)	8933 (8877-9140)
Henan	492 (424-585)	6 (4-8)	25 (16-34)	0.024 (0.015-0.033)	5943 (5905-6080)
Hubei	564 (487-671)	3 (2-4)	11 (7-15)	0.019 (0.012-0.026)	6818 (6776-6977)
Hunan	505 (436-600)	2 (1-3)	8 (5-10)	0.014 (0.009-0.020)	6100 (6061-6241)
Inner Mongolia	742 (640-881)	14 (8-18)	57 (35-76)	0.034 (0.021-0.048)	8959 (8902-9166)
Jiangsu	619 (534-736)	2 (1-3)	9 (5-12)	0.007 (0.005-0.010)	7481 (7434-7655)
Jiangxi	530 (457-630)	10 (6-14)	43 (26-57)	0.058 (0.036-0.081)	6404 (6363-6552)
Jilin	810 (699-963)	13 (8-17)	54 (33-72)	0.024 (0.015-0.033)	9784 (9723-10011)

Liaoning	839 (723-997)	3 (2-4)	13 (8-17)	0.007 (0.004-0.010)	10130 (10067-10365)
Ningxia	598 (516-711)	10 (6-14)	43 (27-58)	0.055 (0.034-0.077)	7226 (7181-7394)
Qinghai	672 (580-798)	22 (14-30)	92 (57-123)	0.098 (0.061-0.137)	8115 (8064-8303)
Shaanxi	596 (514-709)	13 (8-18)	55 (34-73)	0.051 (0.032-0.071)	7202 (7156-7369)
Shandong	534 (461-635)	3 (2-4)	12 (8-16)	0.009 (0.005-0.012)	6454 (6413-6604)
Shanghai	856 (739-1018)	4 (3-6)	18 (11-24)	0.014 (0.008-0.019)	10345 (10280-10585)
Shanxi	639 (551-759)	12 (8-17)	52 (32-69)	0.042 (0.026-0.058)	7718 (7670-7897)
Sichuan	773 (667-919)	7 (4-9)	27 (17-37)	0.039 (0.024-0.054)	9343 (9285-9560)
Tianjin	812 (701-966)	6 (4-9)	27 (17-36)	0.022 (0.014-0.030)	9815 (9753-10042)
Tibet	875 (755-1040)	9 (5-15)	39 (21-63)	0.139 (0.086-0.193)	10568 (10502-10813)
Xinjiang	634 (547-753)	10 (8-17)	44 (33-72)	0.129 (0.080-0.179)	7654 (7606-7831)
Yunnan	851 (736-1000)	13 (8-17)	52 (32-70)	0.069 (0.043-0.096)	10159 (10096-10395)
Zhejiang	781 (674-928)	4 (3-5)	17 (11-23)	0.011 (0.007-0.016)	9435 (9376-9654)

UR Uncertainty range

REFERENCES

1. Lai X, Wahl B, Yu W, Xu T, Zhang H, Garcia C, et al. National, regional, and provincial disease burden associated with Streptococcus pneumoniae and Haemophilus influenzae type b in children in China: modelled estimates for 2010–17. *Lancet Reg Health West Pac* 2022; **22**: 100430.
2. Wahl B, O'Brien KL, Greenbaum A, Majumder A, Liu L, Chu Y, et al. Burden of Streptococcus pneumoniae and Haemophilus influenzae type b disease in children in the era of conjugate vaccines: global, regional, and national estimates for 2000-15. *Lancet Glob Health* 2018; **6**: e744–e57.
3. Zhou M, Wang H, Zeng X, Yin P, Zhu J, Chen W, et al. Mortality, morbidity, and risk factors in China and its provinces, 1990-2017: a systematic analysis for the Global Burden of Disease Study 2017. *Lancet* 2019; **394**: 1145–58.
4. GBD. Global Burden of Disease Study 2019 (GBD 2019) Results. [cited 2022 March 01]. Available from: http://ghdx.healthdata.org/gbd-results-tool.
5. He C, Liu L, Chu Y, Perin J, Dai L, Li X, et al. National and subnational all-cause and cause-specific child mortality in China, 1996-2015: a systematic analysis with implications for the Sustainable Development Goals. *Lancet Glob Health* 2017; **5**: e186–e97.

Webappendix 4. Methods for estimating direct and indirect costs of pneumococcal diseases by province

Direct medical costs of inpatient and outpatient pneumonia, meningitis, NPNM, AOM, and sequelae were estimated using individual-level health insurance data obtained from the China Healthcare Insurance Research Association (CHIRA). In 2008, the Chinese government established a routine reporting system for health service utilization of patients with Urban Basic Medical Insurance, which included available data from three sample cities in each province or municipalities in all 31 provinces of Mainland China. Hong Kong and Macao special administrative regions and Taiwan were excluded from this analysis. We extracted cost data from 2013–2017 from the CHIRA database. The data contained basic demographic information and medical service utility information (e.g., disease diagnosis, medical expenses, and days of hospitalization). CHIRA data were categorized by ICD-10 code, and the ICD-10 case definitions used for this analysis are described in Table 1.

Table 1. ICD-10 codes used for each syndrome

Disease Name	ICD-10 Codes
Pneumonia	B01.2, J09-J18, J20- J21.0, P23.0- P23.9,
Meningitis	A17.0- A17.1, A20.3, A32.1, A39.0, A87, B00.3, B01.0, B02.1, B26.1, B37.5, B38.4, C70, D32, D42, G0, G00, G01, G02, G96.1
NPNM	A02.1, A22.7, A26.7, A32.7, A40.0- A40.3, A41.0- A41.9
AOM	H66, H66.0-H66.4, H66.9, H65, H65.0-H65.4, H65.9, B05.3, H67, H67.0, H67.1, H67.8
Hemiplegia	G81.0, G81.1, G81.9
Hearing Loss	H90- H91
Cognitive Disability	F70-F79, F84.4, Z81.0
Epilepsy	F80.3, G40, T42, Z82.0, X41, X61, Y11

Average inpatient and outpatient direct medical costs for pneumonia and AOM were estimated by province. Meningitis and NPNM direct medical costs were estimated by region due to their small sample sizes (See Table 2). Regional average cost per case estimates were weighted by the expected distribution of cases in each province. The direct medical cost of sequelae was estimated at the national level, and the cost of hearing loss sequelae included the average cost of cochlear implant multiplied by the probability of cochlear implant obtained from published literature. [1, 2] All CHIRA data were analysed using Stata 15 (College Station, TX, USA) and adjusted for

inflation to 2019.

Table 2. Summary of individual-level cost data from 2013 – 2017 obtained from the China Healthcare Insurance Research Association (CHIRA)

Disease	Sample size	Level of Cost Estimates Generated
Inpatient Pneumonia	125,388	Provincial
Outpatient Pneumonia	55,374	Provincial
Meningitis	259	Regional
NPNM	3,177	Regional
AOM	8,864	Provincial
Hemiplegia	69	National
Hearing Loss	221	National
Cognitive Disability	532	National
Epilepsy	2,493	National

Direct non-medical costs of inpatient pneumonia were estimated using data from surveys conducted by the China CDC from 2015 to 2016 in Gansu province.[3] A total of 4,006 children were surveyed, of which 76.4% were from urban areas. Direct non-medical costs of inpatient meningitis were estimated using the national average from another survey conducted in 2014 by the China CDC in Hubei, Shandong and Hebei provinces.[4] Because data on the non-medical cost of NPNM were not available from the surveys by China CDC, the non-medical costs of inpatient NPNM and AOM were estimated by adjusting the non-medical cost of pneumonia by the ratio of inpatient hospitalization days between NPNM/AOM and pneumonia. To generate estimates for all provinces, all non-medical cost estimates were adjusted using the ratio of the provincial total household consumption expenditure obtained from the China Statistics Yearbook. We assumed direct non-medical costs of outpatient pneumonia and AOM only contained the cost of transportation and were equal to the transportation cost of vaccination from the surveys by China CDC.[5]

Indirect costs associated with caregivers/visitors' productivity loss and future lifetime productivity loss due to premature death and disability was estimated using the human capital approach. Caregivers' productivity loss was estimated using the average wage per day of employed persons in urban private units (province-specific wage estimates), average days of hospitalization, and average number of caregivers. The average hospitalized days (lengths of stay) was estimated from the CHIRA database, and the

average number of caregivers for pneumonia and meningitis were assumed to be 1.9 and 2, respectively, based on data from the surveys by China CDC.[3, 4] The number of caregivers for NPNM was assumed to be 1, reaching conservative results given lack of solid evidence. We assumed the same indirect costs among outpatient pneumonia and AOM cases, all of which equalled one half days' productivity loss for 1.9 caregivers. As for visitors' productivity loss, we used the average wage per hour of employed persons in urban private units (province-specific wage estimates) and average visiting hours of visitors per episode. The latter was also obtained from the surveys by China CDC,[3, 4] based on which we used an average visiting hour of 2.7 for all inpatient episodes, including inpatient pneumonia, meningitis, NPNM and AOM cases. We did not account for visitors' productivity loss for outpatient episodes since it was expected to be minimal.

Future costs after the first five years of life included the lifetime productivity loss due to disability and premature death and for cases with meningitis sequelae, the cost of special education. Lifetime productivity loss due to premature death was estimated using the national average annual wage rate for the average productive life years (16 to 60 years) multiplied by the unemployment rate and discounted to the year of vaccination. To estimate the lifetime productivity loss by province, the national lifetime productivity loss was adjusted using the ratio of annual salary in 2019 in each province. For cases with long-term sequelae from meningitis, we assumed the sequelae remained throughout individual's lifetime and reduced lifetime productivity compared to healthy individuals. The lifetime labour force loss of sequalae was obtained by adjusting the child's lifetime labour value using the utility value of different sequelae as weights, also assuming that the age range of a child with labour productivity was 16 to 60 years old, and then estimated the costs using provincial-level average wage of 16-60 years old by age, where future expected income was discounted using the discount rate of 3%. Here we also accounted for standard unemployment using the unemployment estimates from each province. We assumed special education was required for all sequelae included in the model, and 65% of children with meningitis sequelae had to receive special education. Special education cost for each year of primary and secondary school was discounted back to the year of birth, and the annual cost of special education, derived from China Education Statistics Yearbook and China Education Expenditure Statistical Yearbook,[6, 7] was multiplied by the average years of special education (12 years). For children with hearing loss, we also considered cochlear implant cost by assuming that 40% of children suffering from hearing loss had to receive cochlear implant.

Cochlear implant cost was estimated in the 5th year of life, discounted back to the year of birth. The costs per case and lifetime costs are described in Tables 3-6.

Table 3. Inputs for the calculations of indirect costs (US$)

Province	Average Annual Wage	Discounted Cost of Special Education† (Age 6-18)	Discounted Lifetime Productivity	Unemployment Rate*	Household Consumption
Anhui	6259.4	61660.6	93841.2	2.6%	2393.1
Beijing	10747.2	218224.3	161123.9	1.3%	5686.0
Chongqing	7664.9	78177.5	114912.8	2.6%	2719.3
Fujian	7418.8	98944.4	111222.8	3.5%	3228.4
Gansu	5728.4	68225.3	85880.5	3.0%	1993.3
Guangdong	8105.0	134130.8	121511.4	2.3%	3770.8
Guangxi	5807.8	51949.1	87071.8	2.6%	2039.5
Guizhou	6350.1	43314.8	95201.0	3.1%	1970.5
Hainan	6934.1	102688.0	103956.8	2.3%	2340.1
Hebei	5794.0	67212.4	86864.5	3.1%	2345.3
Heilongjiang	4925.8	71282.9	73849.4	3.5%	2366.7
Henan	5580.4	58227.9	83661.9	3.2%	2086.0
Hubei	5643.0	83536.5	84600.4	2.4%	2573.3
Hunan	5618.0	72852.8	84226.8	2.7%	2607.2
Inner Mongolia	5564.6	95159.1	83425.0	3.7%	2878.4
Jiangsu	7497.0	105616.3	112395.8	3.0%	3565.6
Jiangxi	6124.3	63533.8	91816.3	2.9%	2196.7
Jilin	5045.5	97191.2	75642.0	3.1%	2375.0
Liaoning	5416.9	97021.4	81211.0	4.2%	3109.0
Ningxia	5922.5	80895.5	88791.4	3.7%	2332.2
Qinghai	5558.8	89531.0	83338.6	2.2%	2355.4
Shaanxi	5693.2	90792.1	85352.0	3.2%	2263.7
Shandong	7899.2	94940.1	118425.0	3.3%	2625.5
Shanghai	7906.1	192218.3	118529.9	3.6%	6045.7
Shanxi	4823.1	74487.0	72307.3	2.7%	2076.1
Sichuan	6090.4	60219.7	91308.4	3.3%	2458.2
Tianjin	9076.3	124133.7	136073.1	3.5%	4229.9
Tibet	6070.9	142612.4	91014.6	2.9%	1568.0
Xinjiang	6070.9	103824.6	91014.6	2.1%	2292.2
Yunnan	6176.9	70896.0	92604.4	3.3%	1923.2

Zhejiang	7336.5	114076.2	109990.6	2.5%	4114.1

*The unemployment rate was assumed to have a triangular distribution and range of 1.0% - 5.0% for deterministic and probabilistic sensitivity analysis.

†The discounted cost of special education estimated for ages 6-18 years using the societal perspective.

Table 4. Costs of illness (US$) of pneumonia.

Province	Direct cost (Standard Deviation)		Indirect cost (Standard Deviation)		Total cost (Standard Deviation)	
	Per inpatient case	Per outpatient case	Per inpatient case	Per outpatient case	Per inpatient case	Per outpatient case
Anhui	969 (121)	11 (1)	360 (45)	23 (3)	1329 (166)	33 (4)
Beijing	1603 (200)	29 (4)	582 (73)	39 (5)	2185 (273)	68 (9)
Chongqing	905 (113)	26 (3)	380 (48)	28 (3)	1286 (161)	54 (7)
Fujian	1023 (128)	21 (3)	395 (49)	27 (3)	1418 (177)	48 (6)
Gansu	850 (106)	90 (11)	315 (39)	21 (3)	1165 (146)	111 (14)
Guangdong	1247 (156)	19 (2)	442 (55)	30 (4)	1689 (211)	49 (6)
Guangxi	781 (98)	13 (2)	311 (39)	21 (3)	1092 (136)	34 (4)
Guizhou	1270 (159)	91 (11)	546 (68)	23 (3)	1816 (227)	114 (14)
Hainan	1103 (138)	11 (1)	346 (43)	25 (3)	1449 (181)	37 (5)
Hebei	1112 (139)	10 (1)	347 (43)	21 (3)	1459 (182)	31 (4)
Heilongjiang	1245 (156)	24 (3)	342 (43)	18 (2)	1587 (198)	42 (5)
Henan	961 (120)	11 (1)	322 (40)	20 (3)	1283 (160)	31 (4)
Hubei	972 (121)	19 (2)	314 (39)	21 (3)	1285 (161)	39 (5)
Hunan	943 (118)	10 (1)	239 (30)	20 (3)	1182 (148)	31 (4)
Inner Mongolia	1202 (150)	91 (11)	332 (41)	20 (3)	1533 (192)	111 (14)
Jiangsu	1257 (157)	28 (3)	442 (55)	27 (3)	1700 (212)	55 (7)
Jiangxi	825 (103)	6 (1)	320 (40)	22 (3)	1144 (143)	28 (4)
Jilin	1046 (131)	21 (3)	344 (43)	18 (2)	1390 (174)	40 (5)
Liaoning	1285 (161)	24 (3)	345 (43)	20 (2)	1630 (204)	43 (5)
Ningxia	812 (101)	9 (1)	334 (42)	22 (3)	1146 (143)	31 (4)
Qinghai	1132 (141)	91 (11)	403 (50)	20 (3)	1535 (192)	111 (14)
Shaanxi	927 (116)	91 (11)	302 (38)	21 (3)	1230 (154)	112 (14)
Shandong	922 (115)	25 (3)	450 (56)	29 (4)	1371 (171)	54 (7)
Shanghai	1655 (207)	29 (4)	428 (54)	29 (4)	2084 (260)	58 (7)
Shanxi	1035 (129)	11 (1)	321 (40)	18 (2)	1357 (170)	28 (4)
Sichuan	931 (116)	91 (11)	321 (40)	22 (3)	1252 (156)	113 (14)
Tianjin	1434 (179)	28 (4)	472 (59)	33 (4)	1906 (238)	61 (8)

Tibet	965 (121)	91 (11)	411 (51)	22 (3)	1376 (172)	113 (14)
Xinjiang	828 (103)	9 (1)	296 (37)	22 (3)	1123 (140)	31 (4)
Yunnan	802 (100)	161 (20)	373 (47)	22 (3)	1175 (147)	184 (23)
Zhejiang	1244 (155)	18 (2)	398 (50)	27 (3)	1642 (205)	45 (6)

Table 5. Costs of illness (US$) of inpatient meningitis and NPNM.

Province	Inpatient meningitis			Inpatient NPNM		
	Direct cost (Standard Deviation)	Indirect cost (Standard Deviation)	Total cost (Standard Deviation)	Direct cost (Standard Deviation)	Indirect cost (Standard Deviation)	Total cost (Standard Deviation)
Anhui	3774 (472)	777 (97)	4551 (569)	1664 (208)	186 (23)	1850 (231)
Beijing	7201 (900)	1590 (199)	8791 (1099)	2146 (268)	364 (45)	2510 (314)
Chongqing	3996 (499)	1040 (130)	5036 (630)	2192 (274)	306 (38)	2498 (312)
Fujian	5784 (723)	1098 (137)	6881 (860)	1790 (224)	251 (31)	2041 (255)
Gansu	3577 (447)	777 (97)	4355 (544)	2087 (261)	229 (29)	2316 (289)
Guangdong	6097 (762)	1199 (150)	7296 (912)	1869 (234)	274 (34)	2143 (268)
Guangxi	3604 (450)	788 (99)	4392 (549)	2093 (262)	232 (29)	2326 (291)
Guizhou	3564 (446)	862 (108)	4426 (553)	2083 (260)	254 (32)	2337 (292)
Hainan	3744 (468)	860 (108)	4604 (576)	1656 (207)	206 (26)	1862 (233)
Hebei	3747 (468)	719 (90)	4466 (558)	1657 (207)	172 (22)	1829 (229)
Heilongjiang	3759 (470)	611 (76)	4370 (546)	1660 (207)	147 (18)	1806 (226)
Henan	3597 (450)	692 (87)	4290 (536)	1619 (202)	166 (21)	1785 (223)
Hubei	3878 (485)	700 (88)	4578 (572)	1690 (211)	168 (21)	1858 (232)
Hunan	3898 (487)	697 (87)	4595 (574)	1695 (212)	167 (21)	1862 (233)
Inner Mongolia	4088 (511)	755 (94)	4843 (605)	2215 (277)	222 (28)	2438 (305)
Jiangsu	5978 (747)	1109 (139)	7087 (886)	1839 (230)	254 (32)	2092 (262)
Jiangxi	3661 (458)	760 (95)	4421 (553)	1635 (204)	182 (23)	1817 (227)
Jilin	3764 (470)	626 (78)	4390 (549)	1661 (208)	150 (19)	1811 (226)
Liaoning	5715 (714)	801 (100)	6516 (815)	1773 (222)	183 (23)	1956 (244)
Ningxia	3773 (472)	804 (100)	4577 (572)	2136 (267)	237 (30)	2373 (297)
Qinghai	3786 (473)	754 (94)	4541 (568)	2139 (267)	222 (28)	2361 (295)
Shaanxi	3733 (467)	773 (97)	4506 (563)	2126 (266)	228 (28)	2354 (294)
Shandong	5436 (679)	1169 (146)	6605 (826)	1702 (213)	267 (33)	1970 (246)
Shanghai	7409 (926)	1170 (146)	8578 (1072)	2199 (275)	268 (33)	2466 (308)
Shanxi	3592 (449)	598 (75)	4190 (524)	1618 (202)	144 (18)	1761 (220)
Sichuan	3845 (481)	827 (103)	4672 (584)	2154 (269)	243 (30)	2398 (300)
Tianjin	6361 (795)	1343 (168)	7704 (963)	1935 (242)	307 (38)	2242 (280)

Tibet	3332 (416)	824 (103)	4156 (519)	2025 (253)	243 (30)	2268 (283)
Xinjiang	3750 (469)	824 (103)	4574 (572)	2130 (266)	243 (30)	2373 (297)
Yunnan	3537 (442)	838 (105)	4375 (547)	2077 (260)	247 (31)	2323 (290)
Zhejiang	6295 (787)	1085 (136)	7380 (923)	1918 (240)	248 (31)	2167 (271)

Table 6. Costs of illness (US $) of acute otitis media.

Province	Direct cost (Standard Deviation)		Indirect cost (Standard Deviation)		Total cost (Standard Deviation)	
	Per inpatient case	Per outpatient case	Per inpatient case	Per outpatient case	Per inpatient case	Per outpatient case
Anhui	876 (110)	7 (1)	355 (44)	23 (3)	1231 (154)	29 (4)
Beijing	1206 (151)	15 (2)	512 (64)	39 (5)	1719 (215)	54 (7)
Chongqing	755 (94)	15 (2)	361 (45)	28 (3)	1116 (140)	43 (5)
Fujian	901 (113)	15 (2)	354 (44)	27 (3)	1255 (157)	42 (5)
Gansu	627 (78)	15 (2)	270 (34)	21 (3)	897 (112)	36 (4)
Guangdong	959 (120)	15 (2)	386 (48)	30 (4)	1346 (168)	44 (6)
Guangxi	640 (80)	16 (2)	274 (34)	21 (3)	914 (114)	38 (5)
Guizhou	535 (67)	16 (2)	299 (37)	23 (3)	835 (104)	39 (5)
Hainan	921 (115)	7 (1)	393 (49)	25 (3)	1315 (164)	32 (4)
Hebei	855 (107)	6 (1)	328 (41)	21 (3)	1184 (148)	28 (3)
Heilongjiang	813 (102)	8 (1)	279 (35)	18 (2)	1092 (137)	26 (3)
Henan	831 (104)	7 (1)	316 (40)	20 (3)	1148 (143)	27 (3)
Hubei	915 (114)	7 (1)	320 (40)	21 (3)	1235 (154)	27 (3)
Hunan	1043 (130)	7 (1)	318 (40)	20 (3)	1361 (170)	27 (3)
Inner Mongolia	709 (89)	16 (2)	262 (33)	20 (3)	971 (121)	36 (4)
Jiangsu	898 (112)	15 (2)	357 (45)	27 (3)	1255 (157)	42 (5)
Jiangxi	881 (110)	7 (1)	347 (43)	22 (3)	1228 (154)	29 (4)
Jilin	819 (102)	7 (1)	286 (36)	18 (2)	1105 (138)	25 (3)
Liaoning	818 (102)	15 (2)	258 (32)	20 (2)	1076 (135)	35 (4)
Ningxia	662 (83)	16 (2)	279 (35)	22 (3)	942 (118)	37 (5)
Qinghai	600 (75)	16 (2)	262 (33)	20 (3)	862 (108)	36 (4)
Shaanxi	671 (84)	16 (2)	268 (34)	21 (3)	939 (117)	36 (5)
Shandong	800 (100)	15 (2)	377 (47)	29 (4)	1177 (147)	43 (5)
Shanghai	1252 (157)	15 (2)	377 (47)	29 (4)	1629 (204)	44 (5)
Shanxi	790 (99)	7 (1)	273 (34)	18 (2)	1063 (133)	24 (3)
Sichuan	699 (87)	15 (2)	287 (36)	22 (3)	986 (123)	38 (5)
Tianjin	1043 (130)	15 (2)	433 (54)	33 (4)	1476 (185)	48 (6)

Tibet	537 (67)	16 (2)	286 (36)	22 (3)	824 (103)	38 (5)
Xinjiang	702 (88)	16 (2)	286 (36)	22 (3)	988 (124)	38 (5)
Yunnan	597 (75)	16 (2)	291 (36)	22 (3)	888 (111)	38 (5)
Zhejiang	1006 (126)	15 (2)	350 (44)	27 (3)	1355 (169)	42 (5)

REFERENCES

1. Sun B. Investigation on neonatal hearing screening status in Zhengzhou, 2009–2013. *Maternal and Child Health Care of China* 2015; **30**:571–3. (In Chinese)
2. Qiu J, Yu C, Ariyaratne TV, Foteff C, Ke Z, Sun Y, et al. Cost-Effectiveness of Pediatric Cochlear Implantation in Rural China. *Otol Neurotol* 2017; **38**: e75–e84.
3. Ning GJ, Wang XX, Liu SW, Zhu YY, Zhang BL, Zhang XS. Retrospective Investigation on Diseases Burden of Children with Community Acquired Pneumonia under 5 Years in Baiyin City of Gansu Province, 2015-2016. *Chin J Vaccines Immunization* 2017; **23**: 18–21+12. (In Chinese).
4. Liu W. Study on economic burden of bacterial meningitis in China: Chinese Center for Disease Control and Prevention; 2016.
5. Yu W, Lu M, Wang H, Rodewald L, Ji S, Ma C, et al. Routine immunization services costs and financing in China, 2015. *Vaccine* 2018; **36**: 3041–7.
6. Ministry of Education of the People’s Republic of China. China Education Statistics Yearbook 2018. Beijing: China Statistics Press; 2018. p. 176-182.
7. Ministry of Education of the People’s Republic of China. China Education Expenditure Statistical Yearbook 2018. Beijing: China Statistics Press; 2018.

Webappendix 5. Data sources and methods for estimating PCV coverage for each strategy and the cost of immunization delivery per dose by province

PCV13 Availability in China

In China, 7-valent PCV (PCV7) and 13-valent PCV (PCV13) were regulatory approved and made available to Chinese infants in the private market in 2008 and 2016, respectively. In 2008, China's NIP was expanded from 5 vaccines preventing 7 diseases to 14 vaccines preventing 15 diseases, but PCV7 was not included. Later in 2015, PCV7 was pulled from the private market in China due the expiration of import licenses.[1] There are three PCV13 products available in the private market in China for children aged between 2 months-5 years. Two of the available vaccines are manufactured by domestic companies (Walvax Biotechnology Co., Ltd. at the price of US$ 86.67 [RMB 598] per dose; Shenzhen Kangtai Biological Products Co., Ltd. at the price of US$ 68.12 [RMB 470] per dose), and the other is manufactured by a foreign company (Pfizer Inc. at the price of US$ 101.16 [RMB 698] per dose). China currently only publicly funds domestically-produced vaccines, and the prospect of introducing an exceptionally expensive, imported vaccine into the NIP schedule may not be politically feasible. Therefore, the domestic products are more likely to be considered in the NIP because they are domestically produced and have a lower price.

Although a 3+1 schedule is currently used in the private market, no decision has been made on the schedule for the NIP. In this study, the base case modelled a 3-dose schedule (either 2p+1 or 3p+0) following the recommendation of the latest WHO position paper.[2] In scenario sensitivity analyses, we also estimated the cost-effectiveness of a 3p+1 schedule as recommended by vaccine manufacturers[3, 4] to help inform government decision-making.

PCV13 Coverage

Pathogen-specific pneumococcal disease burden was initially estimated assuming no vaccine use, and then adjusted to account for provincial vaccine coverage and the impact of PCV use.

For *status quo* strategy, PCV coverage rates were estimated using the total doses of PCV in 31 provinces, survey results of PCV dose distributions in ten provinces, and child population data by age from National Bureau of Statistics of China and Chinese CDC immunization population database. Chinese CDC does not collect vaccine coverage of PCV as they are not in China's NIP, but each county and/or district in

China (i.e., approximately 3 000 counties and districts) is required to report the number of doses for all vaccines administered to Chinese CDC, including PCV7 and PCV13. We aggregated the number of PCV doses used in each county and/or district to the provincial level. In 2019, we conducted a nationally representative facility-based survey in ten provinces by collecting vaccination records of more than 6 000 children.[5] In the vaccination records, the number of PCV doses received by each respondent was clearly written or printed. We calculated the distribution of doses received (1, 2, 3, or 4 doses) among those with at least one dose in the survey (Table 1). The dose distribution was used to allocate the total number of doses administered in each province to children who received 1, 2, 3, and 4 doses. For those provinces not approached in the survey, we used data from a neighbouring surveyed province to estimate vaccine coverage in view of their similar socioeconomic development status. Specifically, data from Beijing were used for Tianjin; Jilin data were used for Heilongjiang and Liaoning; Shandong data were used for Hebei; Shanghai data were used for Jiangsu and Zhejiang; Guangdong data were used for Fujian and Hainan; Yunnan data were used for Guizhou and Guangxi; Chongqing data were used for Sichuan, Hubei, and Hunan; Gansu data were used for Inner Mongolia, Ningxia, Qinghai, Xinjiang, and Tibet; Henan data were used for Shanxi and Shaanxi; Jiangxi data were used for Anhui. The number of doses was divided by the number of neonates in each province to estimate dose-specific coverage (Table 2). We used the following formula to estimate dose-specific coverage rates at the provincial level:

$$C_{p,j} = \frac{D_p w_j}{N_p j}$$

where $C_{p,j}$ is the coverage for j total doses (1, 2, 3, or 4 doses) in province p. D_p is the total number of doses delivered in province p obtained from China CDC, w_j is the proportion of children receiving j total doses obtained from the survey conducted in ten provinces, and N_p is the number of neonates in the province. We estimated the dose-specific coverage using survey data from only children older than 18 months of age because the minimum age for a child to be fully vaccinated is 18 months old in China. For the 21 provinces not included in the facility-based survey, data from neighbouring provinces with similar levels of economic development were used.

Table 1. PCV dose distribution in the survey of ten provinces

Province	Rural/Urban	0 Dose	1 Dose	2 Dose	3 Dose	4 Dose
Beijing	Rural	0.979	0.007	0.014	0.000	0.000
Beijing	Urban	0.876	0.010	0.010	0.000	0.105

Chongqing	Rural	0.991	0.000	0.000	0.000	0.009
Chongqing	Urban	0.927	0.013	0.009	0.009	0.043
Gansu	Rural	1.000	0.000	0.000	0.000	0.000
Gansu	Urban	0.978	0.011	0.000	0.000	0.011
Guangdong	Rural	1.000	0.000	0.000	0.000	0.000
Guangdong	Urban	0.925	0.009	0.004	0.018	0.044
Henan	Rural	0.980	0.010	0.000	0.010	0.000
Henan	Urban	0.958	0.017	0.000	0.004	0.021
Jiangxi	Rural	0.989	0.000	0.005	0.000	0.005
Jiangxi	Urban	0.935	0.000	0.018	0.012	0.036
Jilin	Rural	0.989	0.011	0.000	0.000	0.000
Jilin	Urban	0.989	0.000	0.005	0.000	0.005
Shandong	Rural	1.000	0.000	0.000	0.000	0.000
Shandong	Urban	0.991	0.000	0.000	0.000	0.009
Shanghai	Rural	0.939	0.037	0.000	0.012	0.012
Shanghai	Urban	0.834	0.008	0.012	0.033	0.112
Yunnan	Rural	1.000	0.000	0.000	0.000	0.000
Yunnan	Urban	0.959	0.005	0.005	0.026	0.005

Table 2. Provincial vaccine coverage in the private market

Province	PCV 1 Dose Coverage	PCV 2 Dose Coverage	PCV 3 Dose Coverage	PCV 4 Dose Coverage
Anhui	0.00000	0.00140	0.00070	0.00244
Beijing	0.01044	0.01392	0.00000	0.07658
Chongqing	0.00360	0.00240	0.00240	0.01320
Fujian	0.00145	0.00073	0.00291	0.00727
Gansu	0.00052	0.00000	0.00000	0.00052
Guangdong	0.00257	0.00129	0.00514	0.01285
Guangxi	0.00038	0.00038	0.00189	0.00038
Guizhou	0.00052	0.00052	0.00259	0.00052
Hainan	0.00168	0.00084	0.00336	0.00840
Hebei	0.00000	0.00000	0.00000	0.00254
Heilongjiang	0.00777	0.00388	0.00000	0.00388
Henan	0.00301	0.00000	0.00120	0.00301
Hubei	0.00176	0.00117	0.00117	0.00646
Hunan	0.00138	0.00092	0.00092	0.00506
Inner Mongolia	0.00005	0.00000	0.00000	0.00005

Jiangsu	0.00195	0.00117	0.00351	0.01092
Jiangxi	0.00000	0.00139	0.00069	0.00242
Jilin	0.00253	0.00127	0.00000	0.00127
Liaoning	0.01153	0.00577	0.00000	0.00577
Ningxia	0.00249	0.00000	0.00000	0.00249
Qinghai	0.00007	0.00000	0.00000	0.00007
Shaanxi	0.00473	0.00000	0.00189	0.00473
Shandong	0.00000	0.00000	0.00000	0.00386
Shanghai	0.01164	0.00698	0.02095	0.06517
Shanxi	0.00090	0.00000	0.00036	0.00090
Sichuan	0.00252	0.00168	0.00168	0.00923
Tianjin	0.00314	0.00419	0.00000	0.02305
Tibet	0.00000	0.00000	0.00000	0.00000
Xinjiang	0.00151	0.00000	0.00000	0.00151
Yunnan	0.00108	0.00108	0.00538	0.00108
Zhejiang	0.00634	0.00380	0.01141	0.03549

For the NIP, the regional 4-dose diphtheria-tetanus-pertussis (DTP) vaccine coverage was used as a proxy for PCV13 coverage because of the similar dosing schedules in China (See Table 3). Regional 4-dose DTP coverage was obtained from a China CDC survey conducted in 2012.[6]

Table 3. Regional dose-specific PCV13 coverage used for the NIP strategy

Dose	East Region<br>Base Case (Range)	Central Region<br>Base Case (Range)	West Region<br>Base Case (Range)
1 Dose	0.0009 (0.0000 - 0.0009)	0.0009 (0.0000 - 0.0009)	0.0009 (0.0000 - 0.0009)
2 Doses	0.0013 (0.0000 - 0.0013)	0.0013 (0.0000 - 0.0013)	0.0066 (0.0000 - 0.0066)
3 Doses	0.0346 (0.0000 - 0.0346)	0.0459 (0.0000 - 0.0459)	0.1458 (0.0000 - 0.1458)
4 Doses	0.9598 (0.8638 - 0.9598)	0.9485 (0.8537 - 0.9485)	0.8433 (0.7590 - 0.8433)

Vaccine coverage was assumed to have a triangular distribution for deterministic and probabilistic sensitivity analysis.

Vaccine Program Costs

The societal cost of the PCV13 program was estimated using regional vaccine program data from a 2015 survey conducted by China CDC in 15 provinces (See Table 4).[7] The governmental cost of routine immunization included the cost of

vaccines, wastage, personnel, cold chain, surveillance, communication activities, training, and supervision at the national and provincial levels. We assumed the wastage rate of PCV13, regardless of the product used, was 5% (range 0-10%) following recommendations from WHO for similar vaccines. The household cost of vaccine-seeking included the cost of transportation and caregiver productivity loss. For the 16 provinces not included in the surveys by China CDC, regional government and household cost estimates were used. The per dose cost of serious adverse reactions was estimated using the cost of abnormal medical examination (US$ 935 or RMB 6,454) from the 2020 China Health Statistical Yearbook,[8] and the incidence of PCV13 adverse events following immunization was obtained from the Adverse Events Following Immunization System (AEFIs) of National Immunization Program Information Management System.[9]

Table 4. Societal cost per dose of the National Immunization Program (NIP) in China in 2015 (2019 US$)

Province or Region	Government Vaccine Program Cost per Dose								Household Cost per Dose	Total Cost per Dose
	Personnel	Office Building	Cold Chain	Surveillance	Communication	Training	Supervision	Other		
Beijing	2.6	0.2	0.2	0.1	0.1	0.0	0.0	0.1	10.2	13.4
Hebei	1.5	0.1	0.3	0.1	0.3	0.1	0.0	0.1	3.7	6.2
Shanxi	1.9	0.1	0.2	0.1	0.1	0.1	0.0	0.0	3.4	5.9
Heilongjiang	2.3	0.1	0.5	0.2	0.5	0.2	0.2	0.2	6.2	10.2
Jiangsu	3.6	0.2	0.2	0.3	0.4	0.1	0.1	0.2	6.1	11.0
Zhejiang	2.8	0.2	0.1	0.2	0.2	0.1	0.0	0.1	6.1	9.8
Anhui	1.6	0.1	0.1	0.1	0.1	0.1	0.1	0.1	4.6	6.9
Jiangxi	1.3	0.1	0.1	0.1	0.1	0.1	0.1	0.0	4.7	6.5
Shandong	1.9	0.1	0.2	0.2	0.2	0.1	0.0	0.1	4.5	7.3
Hunan	2.2	0.1	0.4	0.2	0.3	0.2	0.1	0.1	4.6	8.3
Guangdong	1.6	0.2	0.2	0.2	0.1	0.1	0.0	0.1	6.0	8.6
Guangxi	2.5	0.1	0.2	0.2	0.3	0.1	0.1	0.1	7.7	11.0
Chongqing	2.9	0.1	0.2	0.1	0.2	0.1	0.1	0.1	5.8	9.5
Sichuan	1.9	0.1	0.5	0.1	0.4	0.3	0.1	0.1	5.7	9.3
Gansu	2.9	0.2	0.4	0.2	0.4	0.1	0.1	0.4	3.4	8.1
**East***	2.5	0.2	0.2	0.2	0.2	0.1	0.0	0.1	6.6	10.1
**Central***	1.8	0.1	0.3	0.1	0.2	0.1	0.1	0.1	4.5	7.4
**West***	2.6	0.1	0.3	0.1	0.3	0.2	0.1	0.2	5.6	9.5

**National**	2.2	0.1	0.2	0.2	0.2	0.1	0.1	0.1	5.5	8.8

* For the 16 provinces not included in the China CDC survey, regional routine immunization cost estimates were used.

REFERENCES

1. Bloomberg. Pfizer to cease vaccine operations in China. 2015 [cited 2021 August 11]. Available from: http://www.chinadaily.com.cn/cndy/2015-04/03/content_19988098.htm.
2. WHO. Pneumococcal conjugate vaccines in infants and children under 5 years of age: WHO position paper – February 2019. Geneva: World Health Organization; 2019.
3. Pfizer Inc. Instruction manual for 13-valent Pneumococcal Polysaccharide Conjugate Vaccine. [cited 2021 August 11]. Available from: http://labeling.pfizer.com/ShowLabeling.aspx?id=14515.
4. Walvax Biotechnology Co., Ltd. Instruction manual for 13-valent Pneumococcal Polysaccharide Conjugate Vaccine. [cited 2021 August 11]. Available from: http://www.walvax.com/Info/5/8/index.aspx.

5. Lai X, Rong H, Ma X, Hou Z, Li S, Jing R, et al. Willingness to Pay for Seasonal Influenza Vaccination among Children, Chronic Disease Patients, and the Elderly in China: A National Cross-Sectional Survey. *Vaccines (Basel)* 2020; **8**.
6. Cao L, Wang HQ, Zheng JS, Yuan P, Cao LS, Zhang GM. National Immunization Coverage Survey in China after Integrated more Vaccines into EPI Since 2008. *Chin J Vaccines Immunization* 2012; **18**: 419–424+478. (In Chinese).
7. Yu W, Lu M, Wang H, Rodewald L, Ji S, Ma C, et al. Routine immunization services costs and financing in China, 2015. *Vaccine* 2018; **36**: 3041–7.
8. National Health Commission of China. China Health Statistical Yearbook 2020. Beijing: Chinese Academy of Medical Sciences & Peking Union Medical College Press; 2020.
9. Li K, Zhang L, Ye J, Ji S, Yu W, Cao L. Surveillance of adverse events following immunization in China, 2017. *Chin J Vaccines Immunization* 2020; **26**: 9–18. (In Chinese).

Webappendix 6. Provincial disease burden and economic supplemental results

Table 1. Provincial syndrome-specific cases and deaths averted for the NIP vaccination strategy

Province and Region	Spn Cases Averted								Spn Deaths Averted			
	Inpatient Pneumonia	Outpatient Pneumonia	Meningitis	NPNM	Inpatient AOM	Outpatient AOM	Meningitis Sequelae	Total Cases Averted	Inpatient Pneumonia	Meningitis	NPNM	Total Deaths Averted
Anhui	3983	6880	96	397	633	26872	11	38860	97	12	39	147
Beijing	2008	3469	24	102	322	13672	3	19597	26	3	10	39
Chongqing	2118	3659	40	164	323	13709	5	20013	42	5	16	63
Fujian	2754	4758	57	240	464	19713	7	27986	59	7	24	89
Gansu	2288	3953	136	572	380	16139	16	23469	120	17	56	193
Guangdong	14574	25177	272	1137	2472	105021	32	148653	270	34	112	415
Guangxi	4505	7782	97	394	694	29459	11	42930	119	12	39	169
Guizhou	3427	5921	55	234	544	23089	6	33270	108	7	23	138
Hainan	863	1490	40	164	134	5673	5	8363	57	5	16	78
Hebei	5954	10286	321	1311	879	37331	37	56083	181	40	129	350
Heilongjiang	1418	2450	48	198	210	8905	6	13229	28	6	19	54
Henan	6863	11856	233	957	1065	45253	27	66227	162	29	94	285
Hubei	3564	6158	48	197	554	23542	6	34063	67	6	19	92
Hunan	3681	6359	37	154	577	24491	4	35298	53	5	15	73
Inner Mongolia	1853	3200	94	388	282	11965	11	17781	63	12	38	113

Jiangsu	4859	8394	44	184	771	32748	5	46999	36	6	18	60
Jiangxi	3089	5336	165	678	462	19635	19	29366	179	21	67	266
Jilin	1538	2657	68	277	221	9367	8	14128	37	9	27	73
Liaoning	2765	4776	28	116	430	18282	3	26397	20	3	11	35
Ningxia	699	1208	33	137	110	4660	4	6846	39	4	13	56
Qinghai	613	1060	55	229	96	4079	6	6132	60	7	22	90
Shaanxi	2970	5130	178	741	489	20788	21	30297	151	22	73	246
Shandong	6712	11595	98	421	1137	48309	11	68272	59	12	41	112
Shanghai	1553	2683	21	90	244	10369	2	14961	21	3	9	33
Shanxi	2736	4726	146	603	425	18039	17	26675	114	18	59	192
Sichuan	6934	11979	160	666	1092	46395	19	67226	271	20	65	357
Tianjin	997	1723	21	90	151	6395	2	9376	22	3	9	33
Tibet	528	913	15	64	82	3483	2	5086	73	4	14	92
Xinjiang	1538	2657	70	288	237	10086	8	14877	198	14	44	255
Yunnan	5732	9903	226	961	905	38457	26	56184	397	28	94	519
Zhejiang	4804	8299	66	285	818	34734	8	49005	54	8	28	90
**East**	41027	70875	631	2664	6809	289242	73	411248	566	79	261	906
**Central**	33689	58198	1202	4937	5158	219109	139	322292	974	151	484	1609
**West**	33206	57364	1158	4838	5233	222310	134	324110	1642	152	498	2291
**National**	107922	186437	2992	12438	17201	730661	347	1057650	3182	382	1244	4807

Rows and columns may not sum to the total due to rounding.

Table 2. Provincial discounted economic costs of pneumococcal diseases and 3-dose vaccine program costs (2019 US$) for each vaccination strategy from the societal perspective

Province and Region	Economic Costs of Spn Diseases* (US$ in thousands)			Vaccine Program Costs (US$ in thousands)			Total Costs (US$ in thousands)		
	Status Quo	NIP	Difference	Status Quo	NIP	Difference	Status Quo	NIP	Difference
Anhui	42,164	362	41,802	715	81,598	80,882	42,880	81,960	39,080
Beijing	26,529	6,654	19,875	6,447	34,779	28,333	32,975	41,433	8,458
Chongqing	22,946	2,862	20,084	1,577	39,092	37,516	24,523	41,954	17,431
Fujian	32,423	-24	32,447	1,545	67,052	65,507	33,968	67,028	33,060
Gansu	39,644	7,848	31,796	63	40,056	39,993	39,707	47,904	8,197
Guangdong	180,084	35,711	144,373	9,594	230,080	220,486	189,678	265,791	76,113
Guangxi	44,438	703	43,735	447	93,818	93,372	44,885	94,522	49,637
Guizhou	40,438	1,253	39,185	529	78,606	78,077	40,967	79,859	38,892
Hainan	17,713	3,069	14,644	468	17,564	17,096	18,181	20,633	2,452
Hebei	81,590	11,392	70,198	601	97,523	96,922	82,191	108,915	26,723
Heilongjiang	13,863	1,064	12,799	392	22,184	21,792	14,255	23,248	8,993
Henan	71,794	-4,159	75,953	1,727	155,600	153,873	73,522	151,441	77,920
Hubei	29,160	-2,213	31,373	1,413	70,942	69,528	30,573	68,729	38,156
Hunan	26,857	-5,770	32,627	1,296	84,306	83,010	28,153	78,536	50,383
Inner Mongolia	26,758	5,327	21,431	4	28,289	28,285	26,762	33,617	6,854
Jiangsu	40,512	-1,932	42,444	3,305	101,406	98,101	43,818	99,474	55,657
Jiangxi	53,928	7,491	46,437	540	61,639	61,099	54,468	69,129	14,661

Jilin	16,893	2,772	14,121	117	19,472	19,354	17,010	22,243	5,233
Liaoning	19,727	932	18,795	1,064	40,773	39,709	20,791	41,705	20,914
Ningxia	11,547	1,883	9,664	101	13,715	13,614	11,648	15,598	3,950
Qinghai	16,089	3,871	12,219	2	10,695	10,693	16,091	14,566	-1,526
Shaanxi	51,569	8,491	43,078	1,055	60,957	59,902	52,623	69,448	16,824
Shandong	61,249	-3,430	64,680	1,387	151,579	150,191	62,637	148,148	85,512
Shanghai	17,765	4,040	13,725	4,894	24,774	19,880	22,659	28,814	6,154
Shanxi	36,400	4,468	31,932	155	45,735	45,580	36,554	50,202	13,648
Sichuan	87,047	16,938	70,109	2,990	105,273	102,283	90,037	122,211	32,173
Tianjin	14,203	3,287	10,916	876	14,919	14,043	15,078	18,206	3,127
Tibet	15,401	4,329	11,072	0	7,073	7,073	15,401	11,403	-3,999
Xinjiang	43,789	10,761	33,028	125	28,100	27,975	43,914	38,861	-5,054
Yunnan	106,217	26,356	79,861	1,131	80,882	79,751	107,348	107,238	-110
Zhejiang	47,054	6,140	40,914	9,141	84,729	75,589	56,195	90,869	34,674
**East**	439,546	51,378	388,169	38,254	750,092	711,838	477,800	801,469	323,669
**West**	390,363	18,477	371,886	7,425	656,560	649,135	397,787	675,037	277,249
**Central**	505,883	90,622	415,261	8,024	586,557	578,532	513,908	677,179	163,271
**National**	1,335,792	160,477	1,175,316	53,703	1,993,208	1,939,505	1,389,496	2,153,685	764,189

*Includes the cost of treatment for pneumococcal diseases, pneumococcal meningitis sequelae, and lost productivity due to disability and premature death.

Webappendix 7. Incremental cost-effectiveness ratios of including PCV13 in the NIP for the base case and sensitivity analysis

Table 1. Incremental cost-effectiveness ratios of including 3-dose PCV13 in the NIP

Province and Region	In 2019 RMB			In 2019 US$			Rank
	Cost per Case Averted	Cost per Death Averted	Cost per QALY gained	Cost per Case Averted	Cost per Death Averted	Cost per QALY gained	
Anhui	6,939	1,828,323	59,585	1,006	264,974	8,635	20
Beijing	2,978	1,506,837	47,467	432	218,382	6,879	19
Chongqing	6,010	1,917,929	61,903	871	277,961	8,971	22
Fujian	8,151	2,556,922	82,923	1,181	370,568	12,018	26
Gansu	2,410	292,712	9,768	349	42,422	1,416	6
Guangdong	3,533	1,264,358	40,680	512	183,240	5,896	17
Guangxi	7,978	2,021,535	66,056	1,156	292,976	9,573	24
Guizhou	8,066	1,941,806	64,192	1,169	281,421	9,303	23
Hainan	2,023	217,111	7,283	293	31,465	1,056	5
Hebei	3,288	526,880	17,224	476	76,359	2,496	13
Heilongjiang	4,691	1,157,974	37,387	680	167,822	5,418	16
Henan	8,118	1,886,705	61,339	1,177	273,435	8,890	21
Hubei	7,729	2,868,595	92,352	1,120	415,738	13,384	27
Hunan	9,849	4,753,848	150,597	1,427	688,963	21,826	29
Inner Mongolia	2,660	417,924	13,739	385	60,569	1,991	8

Jiangsu	8,171	6,443,889	193,859	1,184	933,897	28,096	31
Jiangxi	3,445	380,188	12,676	499	55,100	1,837	7
Jilin	2,556	497,976	16,147	370	72,170	2,340	10
Liaoning	5,467	4,144,595	124,974	792	600,666	18,112	28
Ningxia	3,982	484,246	16,178	577	70,181	2,345	11
Qinghai	Cost-saving	Cost-saving	Cost-saving	Cost-saving	Cost-saving	Cost-saving	3
Shaanxi	3,832	472,417	15,715	555	68,466	2,277	9
Shandong	8,642	5,257,431	162,099	1,253	761,947	23,493	30
Shanghai	2,838	1,302,719	41,215	411	188,800	5,973	18
Shanxi	3,530	491,032	16,221	512	71,164	2,351	12
Sichuan	3,302	622,297	20,670	479	90,188	2,996	14
Tianjin	2,301	648,792	21,043	334	94,028	3,050	15
Tibet	Cost-saving	Cost-saving	Cost-saving	Cost-saving	Cost-saving	Cost-saving	1
Xinjiang	Cost-saving	Cost-saving	Cost-saving	Cost-saving	Cost-saving	Cost-saving	2
Yunnan	Cost-saving	Cost-saving	Cost-saving	Cost-saving	Cost-saving	Cost-saving	4
Zhejiang	4,882	2,644,375	82,743	708	383,243	11,992	25
**East**	5,431	2,464,238	78,052	787	357,136	11,312	
**West**	5,936	1,188,783	38,913	860	172,287	5,640	
**Central**	3,476	491,642	16,432	504	71,253	2,381	
**National**	4,985	1,096,931	36,033	723	158,976	5,222	

*The base case analysis assumed 3-dose PCV13 vaccination schedule at the price of RMB 172.5 per dose.

Table 2. Provincial price per dose in NIP at which including 3-dose PCV13 becomes cost-effective

Province and Region	Price in RMB	Price in $US	Rank
Anhui	170	25	22
Beijing	382	55	11
Chongqing	203	29	21
Fujian	219	32	19
Gansu	313	45	15
Guangdong	305	44	17
Guangxi	113	16	27
Guizhou	131	19	26
Hainan	473	69	5
Hebei	303	44	18
Heilongjiang	168	24	23
Henan	161	23	24
Hubei	147	21	25
Hunan	62	9	31
Inner Mongolia	462	67	6
Jiangsu	107	16	28
Jiangxi	390	57	10
Jilin	305	44	16
Liaoning	84	12	29
Ningxia	378	55	12
Qinghai	752	109	3
Shaanxi	444	64	7
Shandong	79	11	30
Shanghai	395	57	8
Shanxi	325	47	14
Sichuan	328	48	13
Tianjin	393	57	9
Tibet	1155	167	1
Xinjiang	862	125	2
Yunnan	574	83	4
Zhejiang	211	31	20
**National**	285	41	-

Table 3. Provincial incremental cost-effectiveness ratios (Cost per QALY gained) of including 3-dose PCV13 in the NIP at different prices*

Province and Region	Cost per QALY gained (Private market price)			Cost per QALY gained (Upper bound recommended by UNICEF for MICs)			Cost per QALY gained (PAHO price)		
	US$	RMB	Rank	US$	RMB	Rank	US$	RMB	Rank
Anhui	27,147	187,318	20	8,635	59,585	20	4,127	28,478	20
Beijing	30,915	213,310	24	6,879	47,467	19	1,026	7,079	17
Chongqing	28,648	197,671	23	8,971	61,903	22	4,180	28,840	21
Fujian	34,767	239,893	25	12,018	82,923	26	6,478	44,696	26
Gansu	8,522	58,804	6	1,416	9,768	6	Cost-saving	Cost-saving	6
Guangdong	23,254	160,449	17	5,896	40,680	17	1,669	11,513	18
Guangxi	26,444	182,465	18	9,573	66,056	24	5,465	37,707	25
Guizhou	27,752	191,491	21	9,303	64,192	23	4,810	33,191	23
Hainan	8,112	55,976	5	1,056	7,283	5	Cost-saving	Cost-saving	5
Hebei	12,092	83,436	13	2,496	17,224	13	159	1,099	10
Heilongjiang	17,983	124,081	16	5,418	37,387	16	2,359	16,275	19
Henan	26,958	186,008	19	8,890	61,339	21	4,490	30,979	22
Hubei	38,712	267,111	26	13,384	92,352	27	7,216	49,792	27
Hunan	58,115	400,993	29	21,826	150,597	29	12,988	89,618	29

Inner Mongolia	10,054	69,372	8	1,991	13,739	8	28	190	9
Jiangsu	74,569	514,526	31	28,096	193,859	31	16,778	115,767	31
Jiangxi	9,862	68,049	7	1,837	12,676	7	Cost-saving	Cost-saving	8
Jilin	11,202	77,291	12	2,340	16,147	10	182	1,256	11
Liaoning	51,069	352,374	28	18,112	124,974	28	10,086	69,595	28
Ningxia	10,331	71,286	9	2,345	16,178	11	400	2,757	14
Qinghai	3,369	23,243	3	Cost-saving	Cost-saving	3	Cost-saving	Cost-saving	3
Shaanxi	10,374	71,579	10	2,277	15,715	9	306	2,110	12
Shandong	65,720	453,470	30	23,493	162,099	30	13,209	91,142	30
Shanghai	28,415	196,062	22	5,973	41,215	18	508	3,505	15
Shanxi	10,713	73,922	11	2,351	16,221	12	314	2,169	13
Sichuan	12,718	87,752	14	2,996	20,670	14	628	4,333	16
Tianjin	16,628	114,732	15	3,050	21,043	15	Cost-saving	Cost-saving	7
Tibet	1,104	7,615	1	Cost-saving	Cost-saving	1	Cost-saving	Cost-saving	1
Xinjiang	3,012	20,784	2	Cost-saving	Cost-saving	2	Cost-saving	Cost-saving	2
Yunnan	5,144	35,497	4	Cost-saving	Cost-saving	4	Cost-saving	Cost-saving	4
Zhejiang	39,483	272,432	27	11,992	82,743	25	5,297	36,548	24
**East**	36,356	250,853		11,312	78,052		5,213	35,970	
**West**	19,279	133,023		5,640	38,913		2,318	15,995	
**Central**	10,750	74,177		2,381	16,432		343	2,369	
**National**	18,622	128,492		5,222	36,033		1,959	13,517	

*Private market price: US$ 68.12; RMB 470. UNICEF MICs upper bound price (base case): US$ 25; RMB 172.5. PAHO price: US$ 14.5; RMB 100.05.

Table 4. Provincial incremental cost-effectiveness ratios (Cost per QALY gained) of including 4-dose PCV13 in the NIP at different prices*

Province and Region	Cost per QALY gained (Private market price)			Cost per QALY gained (Upper bound recommended by UNICEF for MICs)			Cost per QALY gained (PAHO price)		
	US$	RMB	Rank	US$	RMB	Rank	US$	RMB	Rank
Anhui	37,346	257,687	21	12,955	89,390	20	7,015	48,404	20
Beijing	44,353	306,035	24	12,594	86,896	19	4,859	33,530	19
Chongqing	38,493	265,602	22	13,251	91,432	22	7,104	49,016	21
Fujian	47,905	330,547	25	17,845	123,134	25	10,525	72,622	26
Gansu	12,056	83,184	5	2,939	20,278	6	719	4,959	6
Guangdong	33,033	227,929	17	10,097	69,672	17	4,512	31,131	17
Guangxi	35,202	242,893	18	13,559	93,558	24	8,288	57,190	24
Guizhou	37,111	256,065	20	13,443	92,759	23	7,680	52,989	23
Hainan	12,183	84,064	6	2,858	19,724	5	588	4,055	5
Hebei	17,377	119,904	13	4,698	32,413	13	1,610	11,107	12
Heilongjiang	25,165	173,639	16	8,611	59,413	16	4,579	31,595	18
Henan	36,943	254,904	19	13,137	90,644	21	7,339	50,642	22
Hubei	52,658	363,337	26	19,287	133,078	27	11,160	77,003	27
Hunan	78,334	540,503	29	30,520	210,588	29	18,876	130,244	29

Inner Mongolia	14,146	97,608	7	3,803	26,239	8	1,284	8,858	8
Jiangsu	101,642	701,327	31	40,234	277,612	31	25,279	174,424	31
Jiangxi	14,261	98,401	8	3,687	25,443	7	1,112	7,675	7
Jilin	16,108	111,145	12	4,432	30,582	12	1,589	10,962	11
Liaoning	70,132	483,911	28	26,585	183,434	28	15,980	110,259	28
Ningxia	14,373	99,176	9	4,127	28,479	10	1,632	11,262	13
Qinghai	5,369	37,046	3	313	2,157	3	Cost-saving	Cost-saving	3
Shaanxi	14,460	99,775	10	4,074	28,109	9	1,544	10,657	9
Shandong	89,323	616,328	30	33,525	231,323	30	19,937	137,563	30
Shanghai	40,588	280,055	23	10,934	75,446	18	3,713	25,618	16
Shanxi	15,264	105,318	11	4,245	29,293	11	1,562	10,778	10
Sichuan	17,575	121,268	14	5,103	35,211	14	2,066	14,254	15
Tianjin	24,340	167,945	15	6,398	44,147	15	2,029	13,998	14
Tibet	2,425	16,734	1	Cost-saving	Cost-saving	1	Cost-saving	Cost-saving	1
Xinjiang	4,881	33,679	2	149	1,031	2	Cost-saving	Cost-saving	2
Yunnan	7,756	53,516	4	1,147	7,915	4	Cost-saving	Cost-saving	4
Zhejiang	54,801	378,129	27	18,547	127,976	26	9,718	67,056	25
**East**	50,518	348,577		17,433	120,289		9,376	64,695	
**West**	26,816	185,032		8,837	60,973		4,458	30,761	
**Central**	14,986	103,405		4,250	29,326		1,636	11,286	
**National**	25,909	178,773		8,369	57,746		4,098	28,273	

*Private market price: US$ 68.12; RMB 470. UNICEF MICs upper bound price (base case): US$ 25; RMB 172.5. PAHO price: US$ 14.5; RMB 100.05.

Fig 1. Cost-effectiveness acceptability curves of the national 4-dose PCV13 program for the base case and different vaccine price scenarios

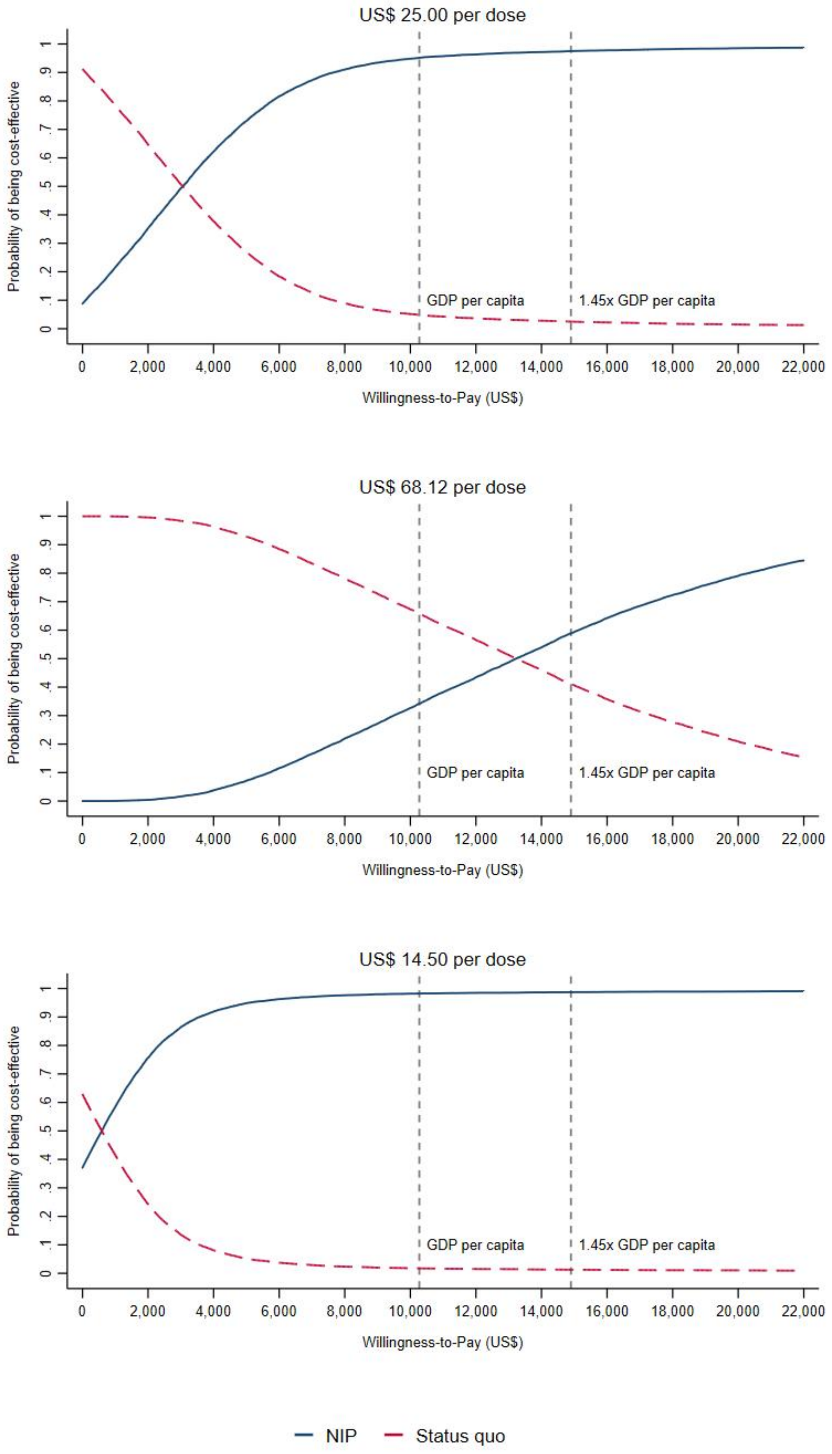

Vertical lines represent the estimated threshold of 1.45x the 2019 national GDP per capita (US$ 14897) by Cai et al. and the 2019 national GDP per capita (US$ 10274). The probability that adding PCV13

into the National Immunization Program is cost-effective for the base case is 95% and increases to 98% when reducing the price of PCV13 to the PAHO price from the probabilistic sensitivity analysis (PSA). The probability decreases to 34% if the price remains at the private market rate.

Fig 2. Cost-effectiveness of 4-dose PCV13 introduction in the national immunization program by region and province for different vaccine price scenarios

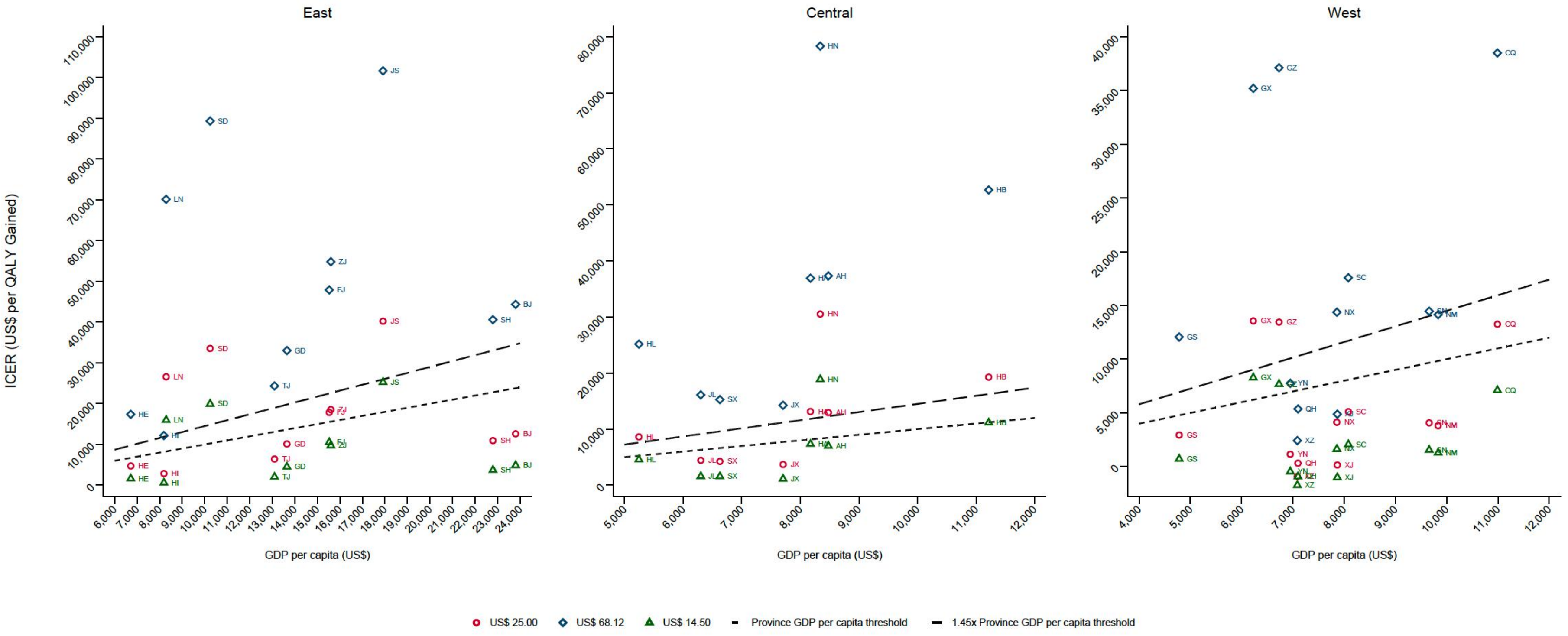

The graphs show ICERs of PCV13 vaccination in the NIP versus *status quo* in the private market by region and province versus the provincial GDP per capita. Different colours and shapes represent the base case and different vaccine price scenarios. The y-axis represents the cost-effectiveness estimate obtained from our model. The dashed

lines represent the cost-effectiveness thresholds of the province GDP per capita and 1.45x the province GDP per capita. ICERs below the lines are cost-effective at that threshold. Credible intervals were omitted for clarity.

Fig 3. Cost-effectiveness acceptability curves of the national 3-dose PCV13 program for different provinces

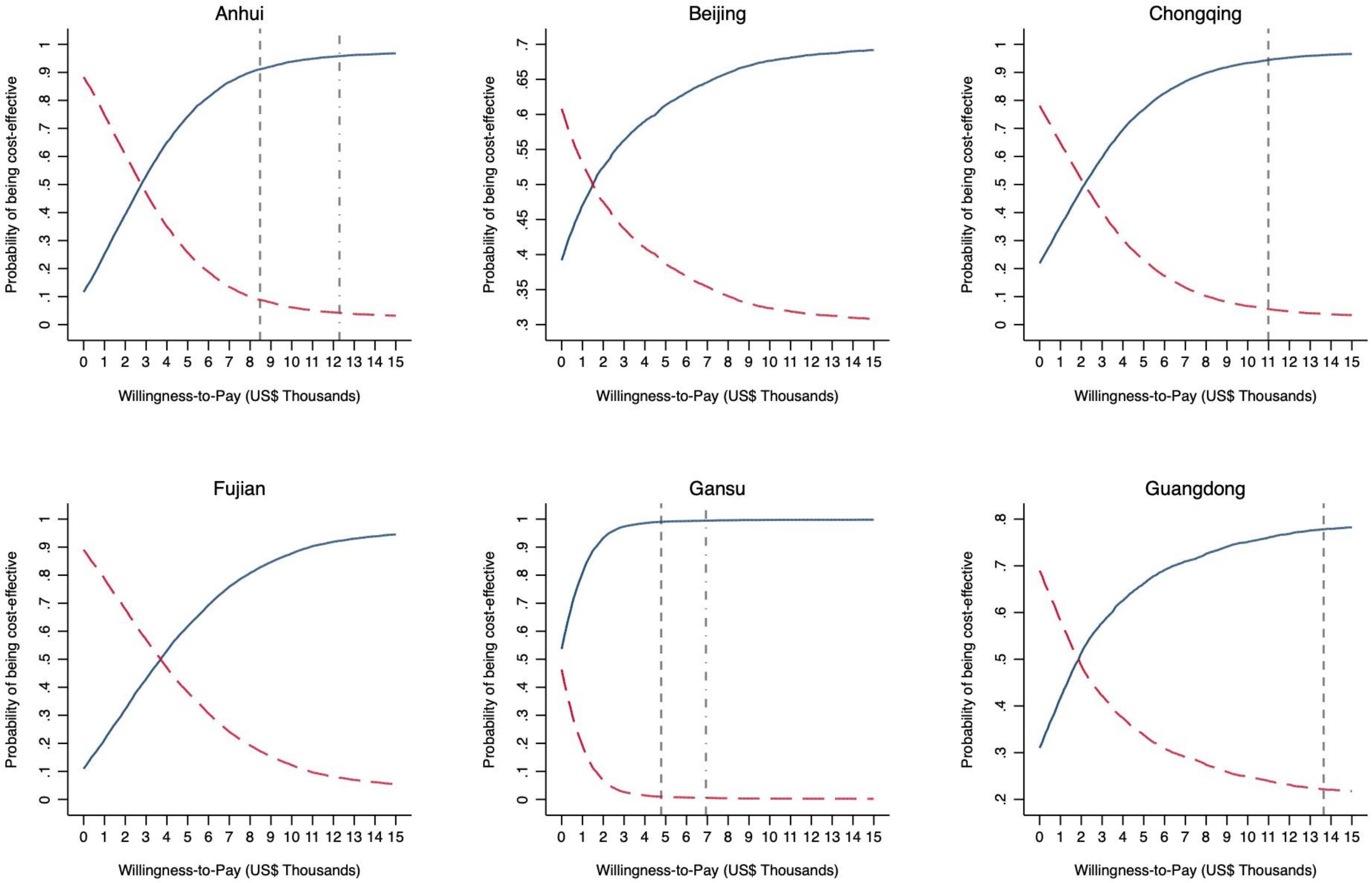
Anhui
Beijing
Chongqing
Fujian
Gansu
Guangdong
Probability of being cost-effective
Willingness-to-Pay (US$ Thousands)
0 1 2 3 4 5 6 7 8 9 10 11 12 13 14 15

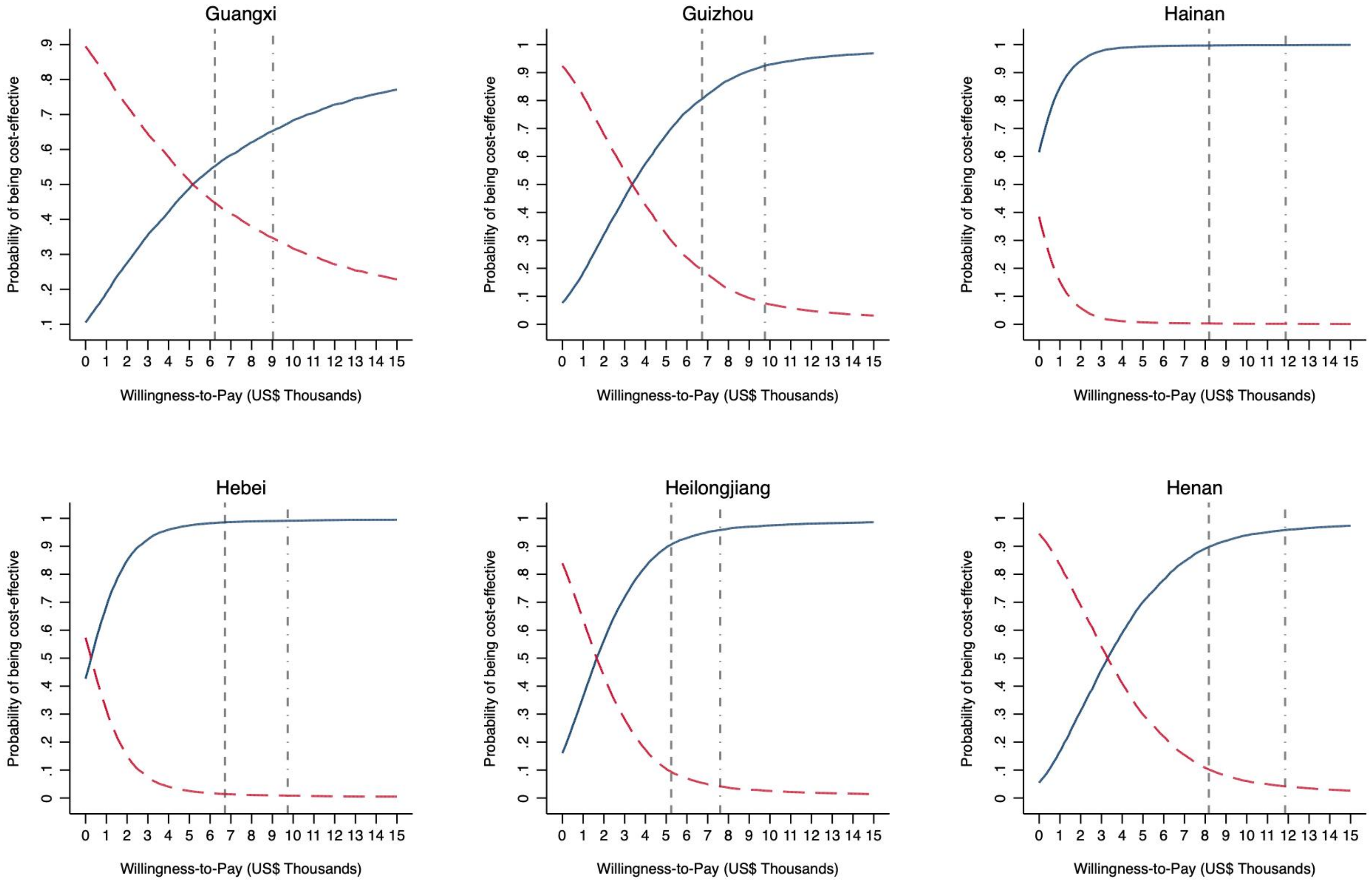
Guangxi
Guizhou
Hainan
Hebei
Heilongjiang
Henan
Probability of being cost-effective
Willingness-to-Pay (US$ Thousands)
0 1 2 3 4 5 6 7 8 9 10 11 12 13 14 15

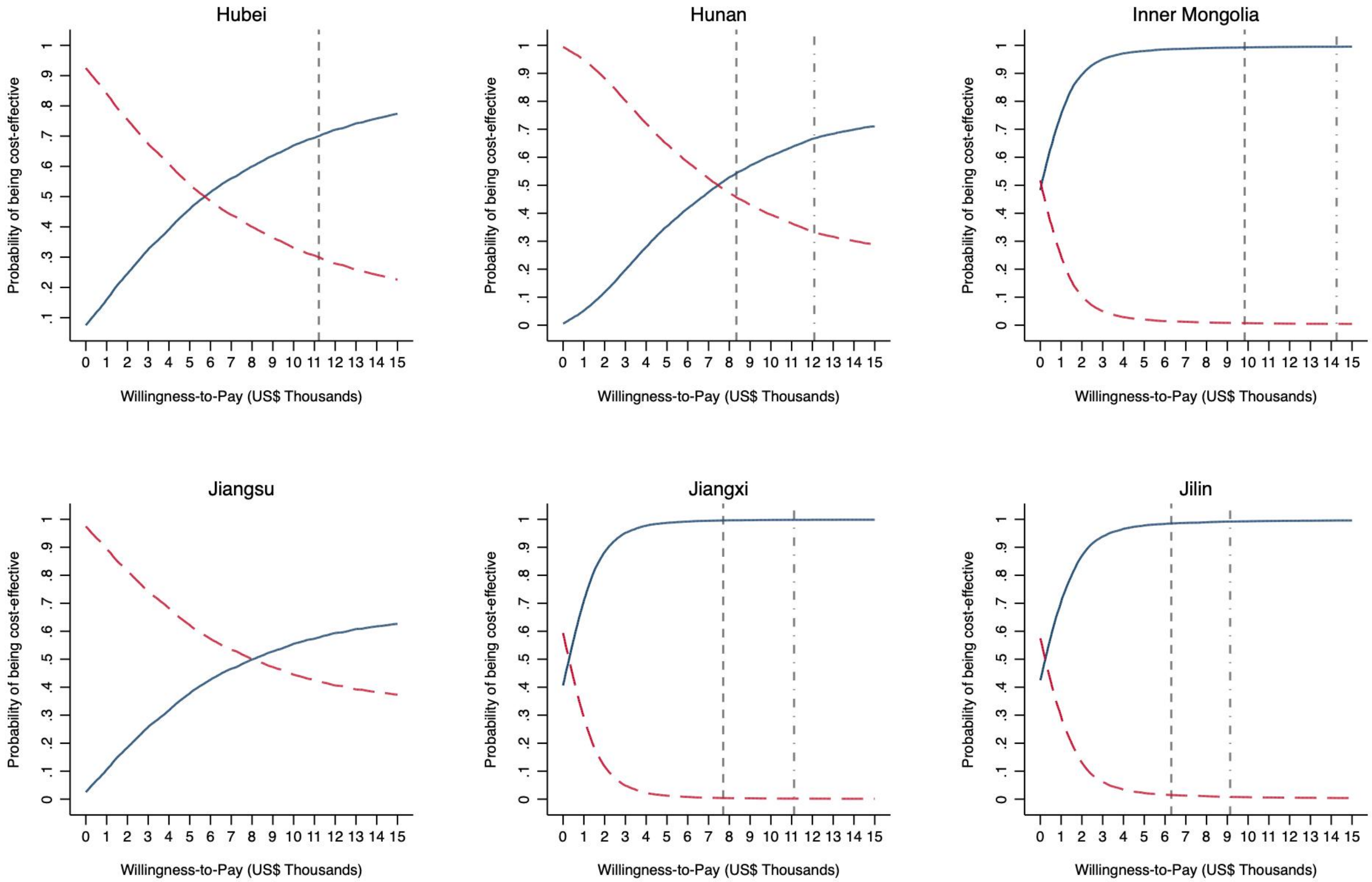
Hubei
Probability of being cost-effective
Willingness-to-Pay (US$ Thousands)
Hunan
Probability of being cost-effective
Willingness-to-Pay (US$ Thousands)
Inner Mongolia
Probability of being cost-effective
Willingness-to-Pay (US$ Thousands)
Jiangsu
Probability of being cost-effective
Willingness-to-Pay (US$ Thousands)
Jiangxi
Probability of being cost-effective
Willingness-to-Pay (US$ Thousands)
Jilin
Probability of being cost-effective
Willingness-to-Pay (US$ Thousands)

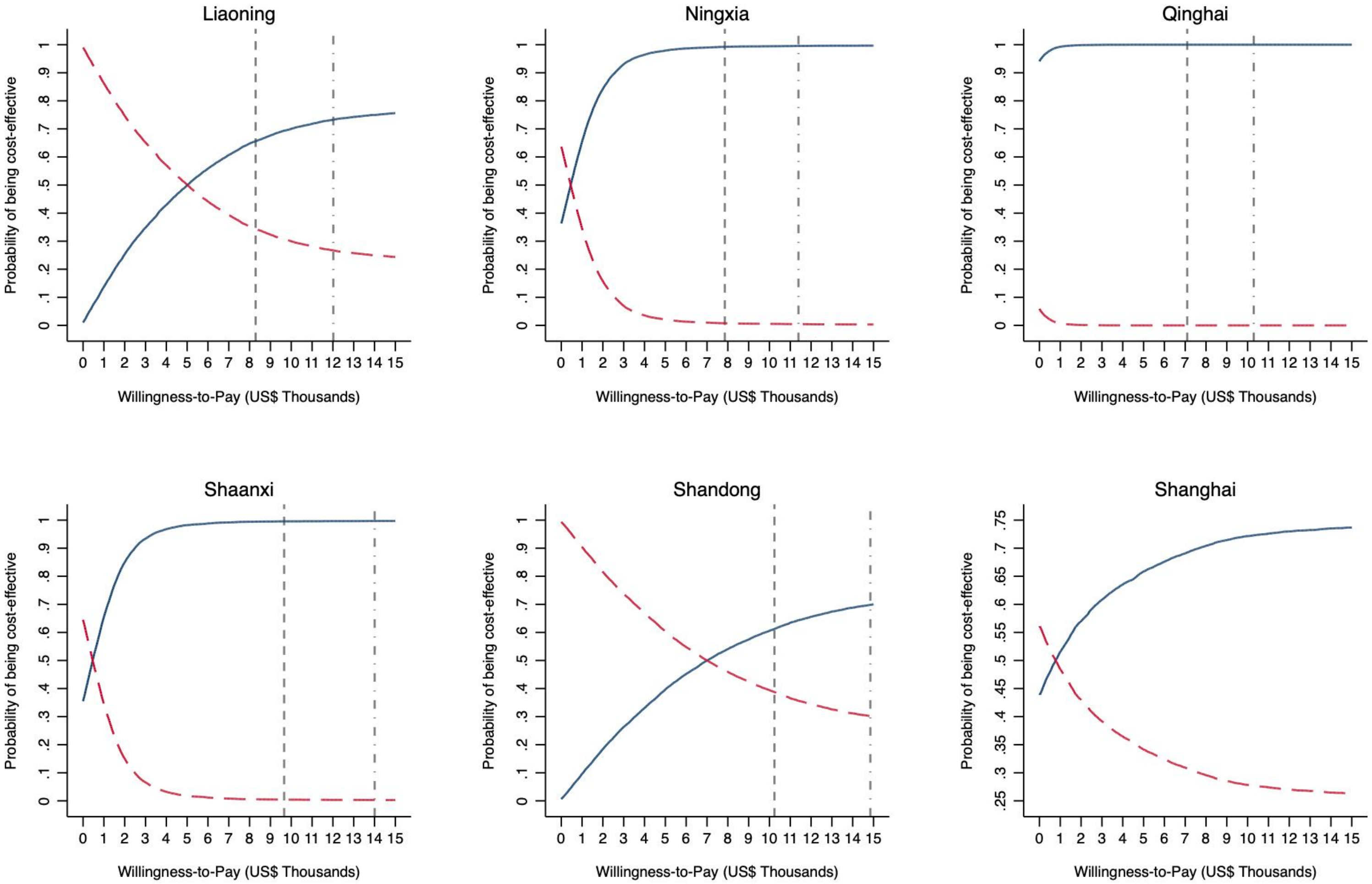
Liaoning
Ningxia
Qinghai
Shaanxi
Shandong
Shanghai
Probability of being cost-effective
Willingness-to-Pay (US$ Thousands)

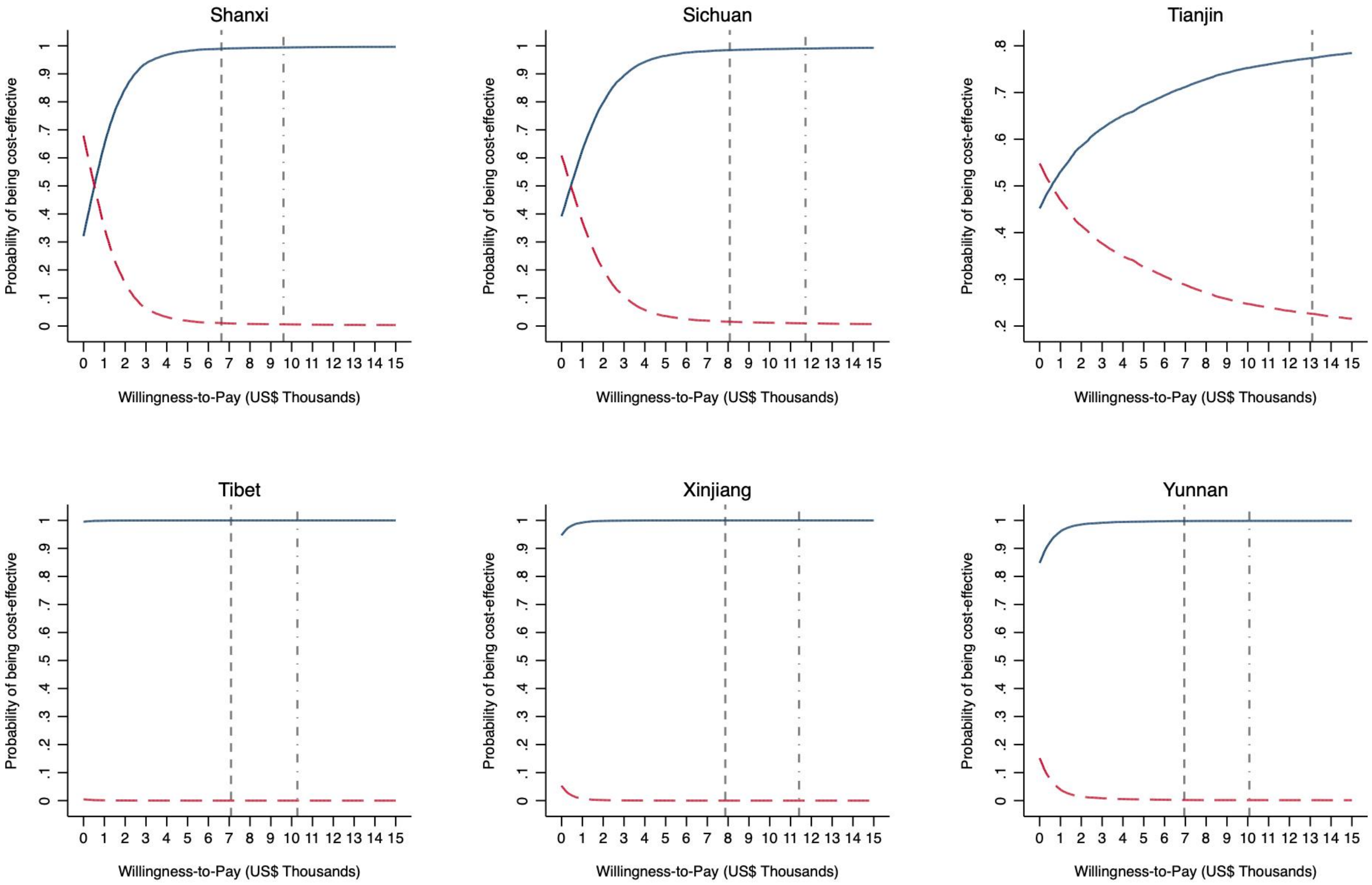
Shanxi
Sichuan
Tianjin
Tibet
Xinjiang
Yunnan
Probability of being cost-effective
Willingness-to-Pay (US$ Thousands)
0 1 2 3 4 5 6 7 8 9 10 11 12 13 14 15
0 .1 .2 .3 .4 .5 .6 .7 .8 .9 1
.2 .3 .4 .5 .6 .7 .8

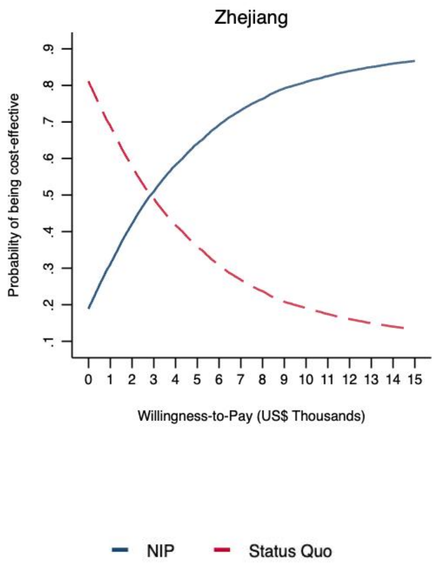

Vertical lines represent the estimated thresholds of 2019 provincial GDP per capita (dash lines) and 1.45x the 2019 provincial GDP per capita estimated by Cai et al (dash-dot lines). Thresholds not displayed are greater than the maximum willingness to pay on the x-axis. Provincial GDP estimates are available in Webappendix 2.

Table 5. Provincial incremental cost-effectiveness ratios (Cost per QALY gained in US$) of including 3-dose PCV13 in the NIP with different IRRs for IPD*

Province and Region	Cost per QALY gained				
	IRR=0.2, assumed min	IRR=0.2534, range min	IRR=0.3334, base case	IRR=0.4386, range max	IRR=0.5, assumed max
Anhui	6,453	7,235	8,635	11,067	12,943
Beijing	3,979	5,012	6,879	10,182	12,784
Chongqing	6,543	7,412	8,971	11,686	13,787
Fujian	9,126	10,162	12,018	15,238	17,729
Gansu	543	856	1,416	2,392	3,148
Guangdong	3,838	4,575	5,896	8,192	9,966
Guangxi	7,316	8,125	9,573	12,088	14,028
Guizhou	6,954	7,796	9,303	11,922	13,944
Hainan	144	470	1,056	2,080	2,878
Hebei	1,386	1,783	2,496	3,738	4,699
Heilongjiang	3,805	4,382	5,418	7,223	8,621
Henan	6,732	7,505	8,890	11,298	13,160
Hubei	10,422	11,484	13,384	16,677	19,213
Hunan	17,502	19,055	21,826	26,609	30,277
Inner Mongolia	958	1,328	1,991	3,146	4,039
Jiangsu	22,270	24,367	28,096	34,493	39,364
Jiangxi	890	1,229	1,837	2,899	3,722
Jilin	1,280	1,660	2,340	3,524	4,440
Liaoning	14,058	15,516	18,112	22,574	25,979
Ningxia	1,320	1,687	2,345	3,493	4,383
Qinghai	Cost-saving	Cost-saving	Cost-saving	Cost-saving	443
Shaanxi	1,246	1,614	2,277	3,436	4,335
Shandong	18,617	20,371	23,493	28,856	32,947
Shanghai	3,559	4,418	5,973	8,731	10,911
Shanxi	1,384	1,730	2,351	3,433	4,271
Sichuan	1,793	2,224	2,996	4,343	5,388
Tianjin	1,376	1,974	3,050	4,928	6,388
Tibet	Cost-saving	Cost-saving	Cost-saving	Cost-saving	Cost-saving
Xinjiang	Cost-saving	Cost-saving	Cost-saving	Cost-saving	273
Yunnan	Cost-saving	Cost-saving	Cost-saving	726	1,295

Zhejiang	8,815	9,952	11,992	15,551	18,312
**National**	3,587	4,172	5,222	7,051	8,467

*Base case value and range for IPD IRR: 0.3334 (0.2534-0.4386).

Table 6. Provincial incremental cost-effectiveness ratios (Cost per QALY gained in US$) of including 3-dose PCV13 in the NIP with different 3-dose coverage rates in the NIP

Province and Region	Cost per QALY gained		
	Base case coverage rates	3-dose coverage rate=90%*	3-dose coverage rate=80%*
Anhui	8,635	7,322	5,947
Beijing	6,879	4,824	2,674
Chongqing	8,971	7,491	5,913
Fujian	12,018	10,247	8,394
Gansu	1,416	907	364
Guangdong	5,896	4,601	3,246
Guangxi	9,573	8,242	6,823
Guizhou	9,303	7,920	6,445
Hainan	1,056	506	Cost-saving
Hebei	2,496	1,834	1,142
Heilongjiang	5,418	4,445	3,426
Henan	8,890	7,596	6,243
Hubei	13,384	11,571	9,674
Hunan	21,826	19,165	16,381
Inner Mongolia	1,991	1,387	742
Jiangsu	28,096	24,377	20,486
Jiangxi	1,837	1,274	686
Jilin	2,340	1,706	1,042
Liaoning	18,112	15,547	12,863
Ningxia	2,345	1,746	1,107
Qinghai	Cost-saving	Cost-saving	Cost-saving
Shaanxi	2,277	1,670	1,023
Shandong	23,493	20,467	17,302
Shanghai	5,973	4,226	2,399
Shanxi	2,351	1,777	1,176
Sichuan	2,996	2,274	1,506

Tianjin	3,050	1,993	887
Tibet	Cost-saving	Cost-saving	Cost-saving
Xinjiang	Cost-saving	Cost-saving	Cost-saving
Yunnan	Cost-saving	Cost-saving	Cost-saving
Zhejiang	11,992	9,863	7,636
**National**	5,222	4,228	3,182

*Assuming 1-dose and 2-dose coverage rates are 0 in the NIP, which are also minimal in the base case coverage rates (<1%).
