# Supplementary material for: Estimating national, regional and provincial cost-effectiveness of introducing childhood 13-valent pneumococcal conjugate vaccination in China: a modelling analysis
Source: Lancet Reg Health West Pac. 2022 Dec 19;32:100666. doi: 10.1016/j.lanwpc.2022.100666 (PMC9918781; doi:10.1016/j.lanwpc.2022.100666)
Supplement: Caption for supplementary material [file mmc2.docx]

**Webappendix List**

- Webappendix 1: CHEERS 2022 Checklist
- Webappendix 2: Data sources for demographic model parameters in economic model
- Webappendix 3: Data sources and methods for estimating pneumococcal epidemiological parameters by province
- Webappendix 4: Methods for estimating direct and indirect costs of pneumococcal diseases by province
- Webappendix 5: Data sources and methods for estimating PCV coverage for each strategy and the cost of immunization delivery per dose by province
- Webappendix 6: Provincial disease burden and economic supplemental results
- Webappendix 7: Incremental cost-effectiveness ratios of including PCV13 in the NIP for the base case and sensitivity analysis
